# Circadian regulation of slow waves in human sleep: Topographical aspects

**DOI:** 10.1016/j.neuroimage.2015.05.012

**Published:** 2015-08-01

**Authors:** Alpar S. Lazar, Zsolt I. Lazar, Derk-Jan Dijk

**Affiliations:** a Surrey Sleep Research Centre, Faculty of Health and Medical Sciences, University of Surrey, Guildford, UK; b John van Geest Centre for Brain Repair, Department of Clinical Neurosciences, University of Cambridge, Cambridge, UK; c Department of Physics, Babes-Bolyai University, Cluj-Napoca, Romania

**Keywords:** Forced desynchrony, Homeostasis, Slope analyses, EEG

## Abstract

Slow waves (SWs, 0.5–4 Hz) in field potentials during sleep reflect synchronized alternations between bursts of action potentials and periods of membrane hyperpolarization of cortical neurons. SWs decline during sleep and this is thought to be related to a reduction of synaptic strength in cortical networks and to be central to sleep's role in maintaining brain function. A central assumption in current concepts of sleep function is that SWs during sleep, and associated recovery processes, are independent of circadian rhythmicity. We tested this hypothesis by quantifying all SWs from 12 EEG derivations in 34 participants in whom 231 sleep periods were scheduled across the circadian cycle in a 10-day forced-desynchrony protocol which allowed estimation of the separate circadian and sleep-dependent modulation of SWs. Circadian rhythmicity significantly modulated the incidence, amplitude, frequency and the slope of the SWs such that the peaks of the circadian rhythms in these slow-wave parameters were located during the biological day. Topographical analyses demonstrated that the sleep-dependent modulation of SW characteristics was most prominent in frontal brain areas whereas the circadian effect was similar to or greater than the sleep-dependent modulation over the central and posterior brain regions.

The data demonstrate that circadian rhythmicity directly modulates characteristics of SWs thought to be related to synaptic plasticity and that this modulation depends on topography. These findings have implications for the understanding of local sleep regulation and conditions such as ageing, depression, and neurodegeneration which are associated with changes in SWs, neural plasticity and circadian rhythmicity.

## Highlights

• Slow waves (SWs) are thought essential for sleep-dependent recovery processes.
• Their amplitude, incidence, frequency and slope reflect synaptic strength.
• Their regulation has been postulated to be independent of circadian phase.
• We report that all characteristics of SWs depend on circadian phase.
• The strength of sleep dependent and circadian modulation depends on topography.

## Graphical abstract

**Figure:**
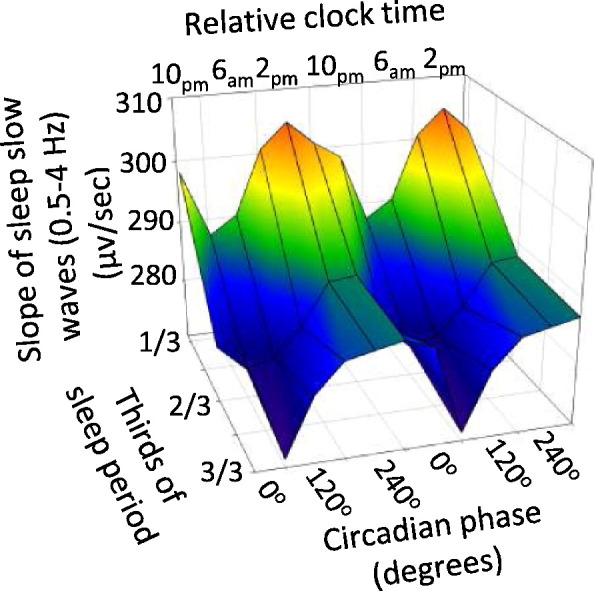

## Introduction

Sleep is thought to reflect a recovery process (Achermann and Borbely, 2003; Daan et al., 1984; Tononi and Cirelli, 2006), the timing of which is gated by the circadian pacemaker located in the suprachiasmatic nucleus (Edgar et al., 1993). The most widely investigated electrophysiological markers of the sleep-dependent recovery process are EEG low-frequency (< 4 Hz), high amplitude (> 75 μV) slow waves (SWs) which have been implicated in sleep-dependent memory consolidation (Rasch and Born, 2013) and age-related changes in cognition (Pace-Schott and Spencer, 2011). The sleep SWs as quantified by visual scoring (Tilley et al., 1987), period amplitude analyses (Uchida et al., 1999) or as slow-wave activity (SWA, EEG power density 0.75–4.5 Hz), decline in the course of sleep and increase in response to the duration and intensity of prior wakefulness (Dijk, 2009; Hung et al., 2013). The sleep-dependent decline in SWs is also observed intra-cortically in humans (Csercsa et al., 2010; Nir et al., 2011). At the cellular level, SWs reflect alternating periods of neuronal activation and silence (Steriade et al., 1993b) and are generated in cortical and thalamo-cortical networks (Steriade et al., 1993a). The characteristics of SWs and the sleep and wake dependent variation in SWs have been investigated in animals (Vyazovskiy et al., 2007), humans (Bersagliere and Achermann, 2010; Nir et al., 2011; Riedner et al., 2007), and large-scale computer models of cortical networks (Esser et al., 2007). It has been concluded that synaptic potentiation during wakefulness and synaptic suppression during sleep are the main mechanisms underlying SW variations. In fact, one leading (Tononi and Cirelli, 2006, 2012, 2014), although challenged (Frank, 2012; Frank and Cantera, 2014) theory, has postulated that SWs are involved in maintaining synaptic homeostasis (Turrigiano, 2008) by reducing synaptic strength, i.e. the number and efficacy of synapses. Furthermore, it is hypothesized that parameters of individual SWs, such as their incidence, amplitude, duration, and the slope, reflect the progression of this process (Nir et al., 2011; Riedner et al., 2007; Vyazovskiy et al., 2007). Although many of these SW parameters change in response to time awake and time asleep, the slope has been demonstrated to be the most sensitive indicator of changes in synaptic strength within neuronal networks (Esser et al., 2007; Riedner et al., 2007; Vyazovskiy et al., 2007).

The change in synaptic strength and associated characteristics of the SWs are presumed to be sleep-dependent and a direct contribution of circadian rhythmicity has been largely ignored. The notion that synaptic plasticity and its marker are primarily sleep-dependent has been based to a larger extent on a comparison of early and late sleep in animals (Vyazovskiy et al., 2007) and humans (Riedner et al., 2007). However, in those comparisons sleep-dependent and circadian factors are confounded because during a 12-hour light period in the rat or an 8-hour sleep period in humans we also traverse 1/2 and 1/3 of a circadian cycle, respectively. Rigorous assessment of a circadian influence on sleep processes requires that sleep is scheduled to many different phases of the circadian cycle, while controlling for the effects of time awake and time asleep. These requirements are met in forced desynchrony protocols which have provided evidence that SWs may be indeed modulated to some extent by circadian rhythmicity (Dijk and Czeisler, 1995), but these findings have not been considered in current models of the regulation of slow waves (Tononi and Cirelli, 2014). This may in part be because in those previous analyses, topographical, i.e. local aspects of slow wave regulation were not considered and the analyses were based on a rather gross measure of slow waves, i.e. spectral power density in the slow wave range. Here, we provide a comprehensive analysis of the circadian modulation of key characteristics of slow waves, and report for the first time that when assessed in a forced desynchrony protocol the incidence, amplitude, duration and in particular slope parameters of SWs during human sleep are to a considerable extent modulated by circadian rhythmicity and that the relative contribution of this circadian effect to the regulation of slow waves, varies widely along the anterior–posterior cortical axis.

## Materials and methods

The study received a favourable opinion from the University of Surrey Ethics Committee and conformed to the Declaration of Helsinki. All participants provided written informed consent before participation in the study. Participants were recruited using advertisements in local newspapers, on local radio and specialized websites. The 271 respondents to the advertisements were screened using multiple sleep, chronotype and health related questionnaires (Lazar et al., 2012). From this pool we selected 36 participants who were able to participate in the experiment during the available time slots and met the stringent inclusion/exclusion criteria. The criteria included consumption of less than 14 units of alcohol per week, no travel across more than 2 time zones during the preceding three months, no shift work, no smoking, and no current medication. Participants were in good mental and physical health as assessed by a standard physical exam, including biochemical profile, full blood count and coagulation screen, and urine analyses for drug of abuse. All participants were free of sleep complaints and underwent a full clinical polysomnography screening to exclude sleep disorders such as sleep apnoea. For more details on recruitment and selection see Hasan et al. (2012).

### Pre-laboratory phase

The study started with a 2-week-long period of monitoring of the habitual sleep–wake cycle by sleep diary and actigraphy (Actiwatch L; Philips Respironics, Best, The Netherlands). The data from the first week of actigraphy were used to calculate the average habitual sleep–wake timing of each participant, whereas during the second week, which occurred immediately preceding the laboratory phase of the study, participants were required to maintain a stable sleep–wake rhythm in accordance with their average habitual schedule. Compliance was monitored by actigraphy.

### Laboratory phase: forced desynchrony protocol

**Fig. 1:**
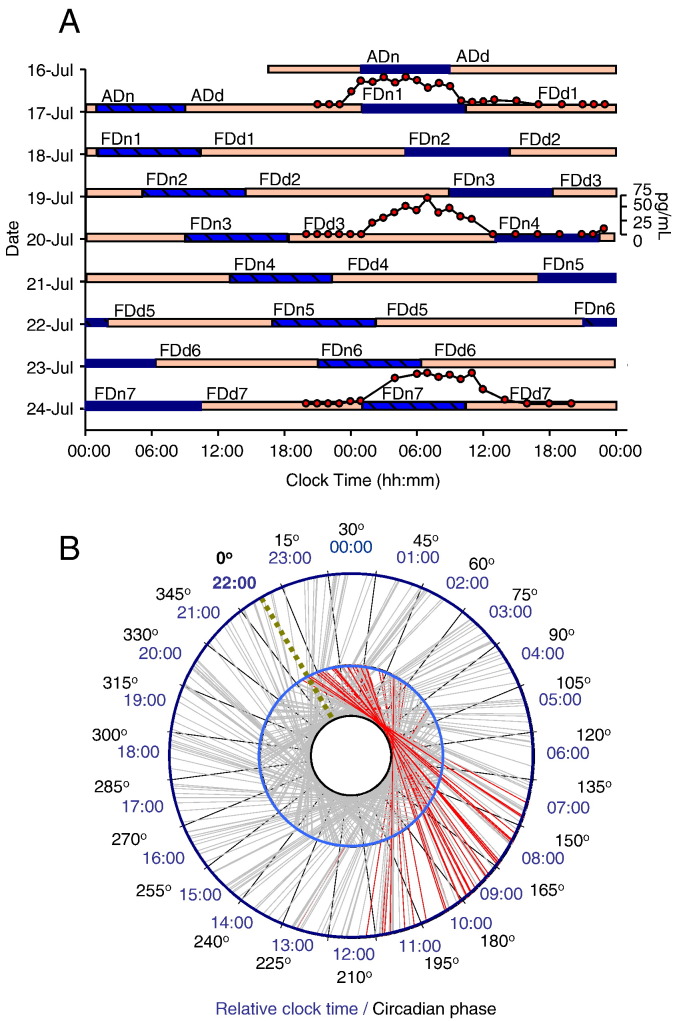
The forced desynchrony protocol. A. Double raster plot of the 28-h forced desynchrony protocol with a representative example of sleep timing and melatonin profile (BB0102, male, 23 years, in vivo circadian period: 24.24 h). Consecutive 24-h periods are plotted next to and below each other. After an 8-hour adaptation night (ADn) followed by an adaption day (ADd) participants were scheduled to a 28-h sleep–wake cycle, in which 9 h and 20 min were scheduled for sleep (blue bars) and 18 h and 40 min for wakefulness (yellow bars). Thus, sleep and wake timing were shifted by 4 h every ‘day’ while at the same time the ratio of sleep and wakefulness remained 1:2, just as during a normal 24-hour day. Melatonin was assessed at baseline (FD1), FD4 and FD7 in order to assess phase and period of the central circadian pacemaker. Blood samples for melatonin concentration assessment were scheduled to be taken hourly but occasionally samples could not be collected due to technical or logistical problems. B. The forced desynchrony protocol represented on a 24-h angular plot indicating each individual baseline (FDn1) sleep period (red lines) against the circadian phase and relative clock time, which corresponds to the habitual sleep timing, duration and circadian phases covered by usual sleep studies. Grey lines indicate all sleep periods scheduled across the circadian cycle (N = 231) during the 10-day protocol for the 34 participants. Each sleep period indicates the time course between the scheduled bedtime (internal light blue circle) and wake-up time (external dark blue circle). The dotted green line indicates the average clock time at circadian phase zero, corresponding to the timing of the dim light melatonin onset.

The forced desynchrony protocol lasted for 10 consecutive days and was modified from a protocol described previously (Dijk and Czeisler, 1995) (Fig. 1). During this time period, all participants were resident in the sleep and circadian research unit of the Surrey Clinical Research Centre of the University of Surrey. After a baseline assessment, the sleep–wake cycle was scheduled to 28 h, of which 9 h 20 min were spent in bed in darkness followed by 18 h 40 min of scheduled wake periods in a dim light environment (< 5 lx), with no access to information about clock time. During the forced desynchrony, consecutive sleep periods were scheduled 4 h later every ‘day’ (Fig. 1A). Because under these conditions the central circadian pacemaker cannot adjust to a 28-h period it ‘free-runs’ and scheduled sleep episodes will occur at all phases of the endogenous circadian rhythm as assessed from plasma melatonin. The rhythm of plasma melatonin is driven by the Suprachiasmatic Nucleus (SCN) of the anterior hypothalamus through a well characterized multi-synaptic neural pathway from the SCN to the pineal and is therefore considered a reliable marker of the phase of the central circadian pacemaker (Pevet and Challet, 2011) that drives the circadian modulation of sleep in primates (Edgar et al., 1993). Under the conditions of this protocol it oscillates with a period of 24.14 ± 0.25 SD h (Hasan et al., 2012).

When participants are sleeping at their habitual nocturnal sleep times, they traverse approximately 1/3 of a circadian cycle from the beginning to the end of sleep and only a very limited part of the entire circadian cycle is covered (Fig. 1B). By contrast, sleep episodes during forced desynchrony occurred at many phases of the endogenous circadian cycle. Thus, any influence of endogenous circadian phase on sleep parameters could be assessed separately from the sleep-dependent modulation (Dijk and Czeisler, 1995).

### Standardization of wake episodes

Because SWA during sleep is considered a use-dependent process (Krueger, 2011; Tononi and Cirelli, 2014), such that SWs during sleep are determined by the duration and intensity of waking (i.e the activities within waking) we standardized both the duration and activities within waking. Participants were continuously monitored during wakefulness by a member of staff to ensure wakefulness and followed a strict schedule without exercise. Meals were scheduled and standardized. In addition the participants completed a performance test battery (duration approximately 40 min) 5 times evenly distributed across the waking episodes. This test battery assessed a variety of cognitive domains, including vigilance, sustained attention, working memory, effort, motor function, etc., and is identical to the test battery described previously (Lo et al., 2012).

### The assessment of the circadian phase

During forced desynchrony period 1 (FD1), FD4, and FD7, blood samples were scheduled to be taken hourly for 28 h in order to assess plasma melatonin (Fig. 1A). Melatonin levels were quantified using radioimmunoassay (Stockgrand Ltd, Guildford, UK). Circadian phase zero was assigned to the time point when the plasma concentration of melatonin reached 25% of the range of the melatonin rhythm [dim light melatonin onset (DLMO)] (Klerman et al., 2002; Lewy et al., 1985). The range of the melatonin rhythm was determined as the maximum melatonin concentration in the actual 24-h sampling period minus the baseline values (values observed during the day), with the melatonin maximum value being the median of the 3 highest values. The circadian period was computed from the linear regression fitted to the 3 DLMOs for each participant (τ = 24 h + slope) as described earlier by us and others (Hasan et al., 2012; Pittendrigh and Daan, 1974). The same analysis method has been applied to assess circadian period in non-laboratory conditions, i.e. free-running blind individuals (Lockley et al., 1997).

### Polysomnographic (PSG) assessment

Complete EEG data for sleep structure and quantitative EEG (qEEG) analyses were obtained from 34 (18 females, age: 25.1 ± 3.4, Morningness–Eveningness Questionnaire score: 49.6 ± 8.4) (Horne and Ostberg, 1976) of the 35 subjects enrolled in the laboratory phase and included in the analyses (231 sleep episodes in total). One participant was excluded after the adaptation (ADn) (Fig. 1A) night of the forced desynchrony protocol for health reasons.

During the adaptation night (ADn) (Fig. 1A), which also served as a clinical sleep screening, a full clinical EEG-PSG (12 EEG channels, EOG, EMG, thoracic belt, a nasal airflow sensor, a microphone, and leg electrodes) was recorded. During the experimental phase of the study, a basic polygraphic (EMG, ECG) with extended monopolar EEG montage was used in order to cover all main brain areas (Fp1, Fp2, F3, F4, C3, C4, T3, T4, P3, P4, O1, and O2) according to the international 10–20 system. The ground and common reference electrodes were placed at FPz and Pz, respectively. Two reference setups were used: common reference (where all EEG derivations were referenced to Pz) and contralateral mastoid reference (where the active EEG derivations were offline re-referenced to the mastoid derivation [A1 and A2] from the contralateral hemisphere). The common reference was used as a default across all analyses robustness of which was verified by using the contralateral mastoid reference. The PSG data were recorded on Siesta 802 devices (Compumedics, Abbotsford, Victoria, Australia). EEG data were stored at 256 Hz. The low-pass filter was set at 70 Hz and the high-pass filter was set at 0.3 Hz. Electrode impedance was kept below 5 kΩ. In accordance with previous forced desynchrony protocols (Cajochen et al., 2002; Dijk, 1995) sleep staging was performed in 30 s epochs according to the Rechtschaffen and Kales criteria (Rechtschaffen and Kales, 1968) by one experienced sleep researcher (ASL) with over 10 years of experience in scoring sleep. In accordance with the standard operating procedures of the Surrey Clinical Research Centre the scoring of ASL was compared to a standard scored data set which showed a concordance exceeding 90%.

### SW detection and analysis

**Fig. 2:**
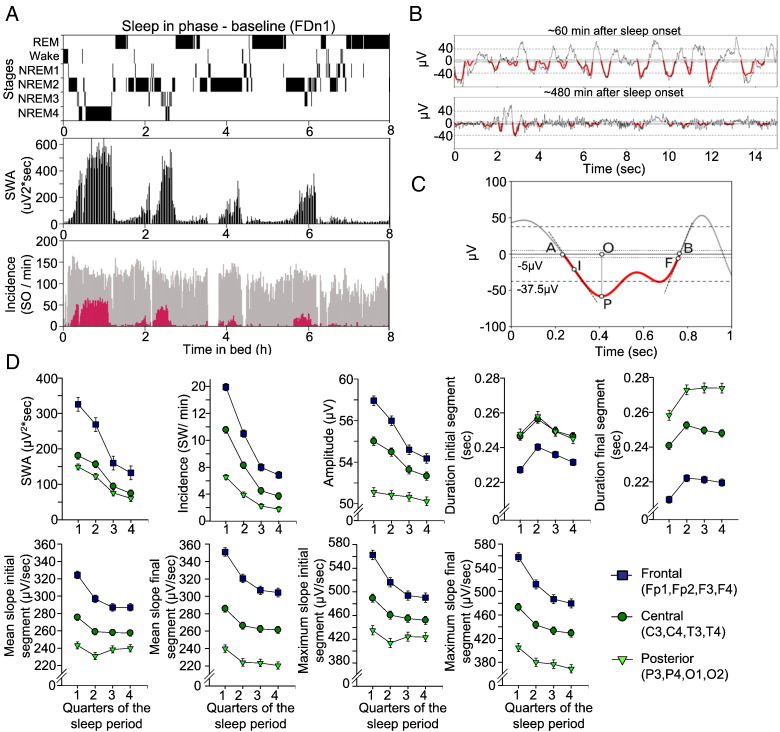
Baseline sleep and SW detection. A. Time course of sleep stages during the participant's (BB0214, Female) baseline night (FDn1) (upper panel), the time course of slow wave activity (SWA: 0.5–4.0 Hz) (middle panel) and the incidence of the individually detected slow waves (SWs) (bottom panel). The grey bars represent all the SWs > 5 μV. The red bars represent the SOs > 37.5 μV. Artefact segments appear as gaps in activity. B. A representative 15-second example of NREM sleep during baseline sleep (FDn1) of the same participant at ~ 1 h (upper panel) and at ~ 8 h after sleep onset, respectively. The thin black line represents the original raw EEG signal, the bold red lines indicate negative half-waves of EEG signals filtered between 0.5 and 4.0 Hz (see Materials and methods). C. Schematic representation of detection of SWs and extraction of relevant properties from a single half-wave in a 0.5–4Hz band-pass filtered signal. The negative half-wave enclosed between zero crossings A and B has its main peak (P) at time O. The dotted line tangential to the wave in I has the steepest slope (maximum slope) in the initial (AO) phase, while the one tangential to the wave in F has the steepest slope in the final (OB) phase. Mean initial and final slopes are calculated as the ratios PO/OA and PO/BO, respectively. The numbers of peaks, two in the example given, are also counted. The dashed lines indicate the ± 5 μV and ± 37.5 μV voltage threshold levels used as amplitude criteria for detecting SWs. D. The effect of time in bed (quarter of sleep period) and brain topography on SWA in NREM sleep and the raw SW measures during the first 8 h of baseline sleep (FDn1). The results for negative half-waves are presented. Least square (Ls) means (absolute values) and standard error of the mean (SEM) derived from the mixed model analyses are indicated for all studied sleep intervals (2 hourly bins) and main brain regions. The brain topography factor comprises three main brain regions each including weighted averages over the Frontal (Fp1, Fp2, F3, F4), Central (C3, C4, T3, T4), and Posterior (P3, P4, O1, O2) areas. The three brain derivations are indicated with different circles (Frontal derivation = dark blue square; Central derivation = dark green circle; Posterior derivation = light green triangle). For statistical results of the SW parameters please refer to Table 1.

After visual scoring, visual identification of EEG artefacts, and appropriate band-pass filtering (see below) of artefact free NREM sleep stages 2, 3 and 4 EEGs from 12 derivations, we applied algorithms based on the NumPy, SciPy and Matplotlib libraries for scientific computing (Hinard et al., 2012) to quantify parameters of SWs such as incidence, amplitude, period, slope in accordance with previous studies (Figs. 2A–C). Channels were analysed independently.

**Fig. S1:**
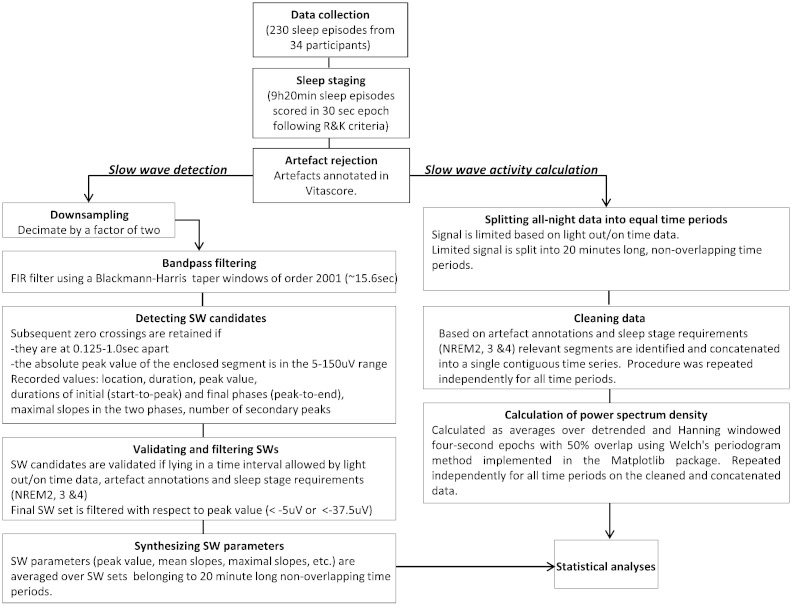
Data processing flow chart from data collection to statistical analysis. The 12 channels were processed independently. The 20 min intervals were the basic units for the statistical analyses of the SW parameters and SWA. The data from these intervals were collapsed into larger sleep and circadian bins depending on the actual statistical analyses. See Materials and methods section for further details.

All EEG artefacts (muscle activity/sweating) for each individual EEG channel were visually identified by an experienced scorer and annotated on a three second basis using the EEG browser software Vitascore version 1.5 (Temec Instruments B.V., Kerkrade, The Netherlands). Thereafter all EEG channels were exported for further qEEG analyses. Segments annotated as artefacts were not used in the subsequent SW detection (for a flowchart of the analyses see Inline Supplementary Fig. S1).

All EEG artefacts (muscle activity/sweating) for each individual EEG channel were visually identified by an experienced scorer and annotated on a three second basis using the EEG browser software Vitascore version 1.5 (Temec Instruments B.V., Kerkrade, The Netherlands). Thereafter all EEG channels were exported for further qEEG analyses. Segments annotated as artefacts were not used in the subsequent SW detection (for a flowchart of the analyses see Inline Supplementary Fig. S1).

Inline Supplementary Fig. S1

Inline Supplementary Fig. S1 can be found online at http://dx.doi.org/10.1016/j.neuroimage.2015.05.012.

The EEG time series were decimated by a factor of two (keeping every second value) and band-pass filtered in the 0.5–4 Hz interval by a FIR window filter using a Blackman–Harris window of 2001 points (~ 15.6 s)(Harris, 1978). The large width of the window, unlike second type Chebyshev filters, has the benefit of producing practically no side lobes in the frequency response of the filter while phase information remains intact (Lyons, 2010). The applied filter was chosen from a number of candidates based on the visual inspection of the filtered signal and its power spectrum.

The main rationale for decimating data prior to filtering was the high computing demand due to the large taper window of the applied FIR filter. Since decimation affects only the highest frequencies (64–128 Hz) while those of interest are in the lowest region of the spectrum (0.5–4 Hz), the results should not be influenced by the procedure. Unless stated otherwise, all quantities introduced below are defined in the context of the filtered signal. Half-waves, i.e. segments enclosed between subsequent zero crossings (points A and B in Fig. 2C), with maxima (minima) values exceeding (below) + 5 μV (− 5 μV), below (exceeding) + 100 μV (− 100 μV), with frequencies in the 0.5–4 Hz interval, not overlapping with segments labelled as artefacts and not belonging to sleep stages other than NREM stages 2, 3 and 4 were retained and a number of properties were extracted following the procedure described previously (Bersagliere and Achermann, 2010; Riedner et al., 2007). The peak of the positive (negative) half-wave is defined as its highest (lowest) point or maximum (minimum) (P). We refer to the segment between the start (A) of the half-wave and its peak location (O) as the initial phase (AO), while the remaining segment (OB) as the final phase. Although we quantified the duration, the frequency of a half-wave can be defined as the inverse of its period, i.e., twice the duration of the half-wave. The mean initial slope is calculated as the half-wave maximum (minimum) divided by the duration of the initial phase (PO/OA). Similarly, the mean final slope refers to the ratio of the maximum (minimum) over the duration of the final phase (PO/BO). The maximum initial slope is defined as the largest absolute value of the signal's first derivate in the initial phase (at I). The maximum final slope is computed similarly from the final phase (at F). Further to these primary SW parameters, we also calculated the duration for the entire SW between the subsequent zero crossings (AB), the overall mean slope and maximum slope as averaged between the initial and final segment of the SW half-waves. The parameters of all individually detected SWs were collapsed into 20 min consecutive intervals between lights out and lights on (28 intervals in 9 h 20 min sleep).

### SWA calculation

All artefact-free EEG segments of the sleep stages of interest (NREM stages 2, 3 and 4) were concatenated within consecutive 20 min intervals between lights out and lights on and power values were calculated as averages over detrended, Hanning windowed four-second epochs with 50% overlap using Welch's periodogram method implemented in the Matplotlib package (Evans et al., 2013).

### Statistics

The basic units used in the statistical analyses were the 30 s epochs for the sleep data (TST, NREM and REM sleep), and the 20 minute intervals for the SW parameters and SWA. These units were then averaged into larger circadian and sleep dependent bins for each sleep period which were entered into the mixed model analyses of variance (see below). The basic 20 minute time intervals used for SW parameters and spectral data was used as an optimal time length worked out empirically allowing sufficient artefact free NREM data within an interval to minimize variance of studied SW parameters and spectral values between the intervals but also sufficient time resolution to allow alignment with the larger circadian and sleep dependent bins which were included in the statistical analyses.

For all our analyses we used a repeated measures design implemented in a mixed model ANOVA including multiple within participant factors and no between participant factors. For an assessment of baseline sleep we analysed the first 8 h of FDn1 (Fig. 1) to allow comparisons with published baseline data. We collapsed the data within 2-hourly intervals (quarters of the sleep period) and across EEG channels over three main brain regions (Frontal: Fp1, Fp2, F3, F4; Central: C3, C4, T3, T4 and Posterior: P3, P4, O1, O2). We entered both factors ‘quarters of sleep period’ and ‘brain regions’ in the same mixed model ANOVA. For the circadian analyses, we used the entire 9 h 20 min sleep episodes across FDn1 to FDn7. We assigned a circadian melatonin phase and time since start of sleep episode to each 30-s epoch and 20 min consecutive interval between lights out and lights on. In the next step data from the sleep epochs (Total sleep, NREM sleep, REM sleep) and the consecutive 20 minute intervals (SW parameters and, spectral data) were averaged in 6 circadian phase bins, each of 60° (~ 4-hourly bins), and three sleep dependent time bins (around 186.7 min each) for each sleep period, similar to published methodology (Dijk et al., 1997).

Mixed model analyses of variance with within participant factors ‘circadian phase’ (6 levels) and ‘time since start of sleep episode’ (3 levels) (sleep dependent factor) and their interaction were computed for all sleep parameters. Two types of covariance structure were used: unstructured and compound symmetry. Using the same mixed models, we performed analyses in which we controlled the circadian and sleep-dependent effects for further within factors such as brain regions, amplitude threshold or frequency range for SW detection, EEG reference, polarity of the half-waves, and segment (initial versus final) of certain SW parameters (duration and slope). In all these additional analyses we also investigated the interaction between the circadian and sleep dependent factors and the additional control factors to assess whether the circadian and sleep dependent modulation were differentially influenced by these factors. We also controlled for the effect of REM and NREM sleep duration on the studied variables by adding it as continuous measure to the same mixed model. The distribution of residuals was assessed for normality for each single analysis. In order to visualize statistical effect sizes, we calculated the Cohen's *f^2^* effect size (Cohen, 1988):

$$f^{2}=u/v*F\text{,}$$

where *u* and *v* are, respectively, the numerator and denominator degrees of freedom of the F statistic used to determine the corresponding main or interaction effect in the general linear mixed model analysis. In all cases, the averaging of SW parameters within the studied time bins and/or brain regions was weighted for the numbers of detected SWs in that particular time bin or brain region. The target SW measures such as the slope characteristics were highly correlated (Inline Supplementary Table S1) with each other and with the amplitude and the duration. This argues against the application of a conservative correction for multiplicity such as Bonferroni. However, in order to minimize the chance of type I error we set alpha at *P* < 0.005. All statistical analyses were performed in SAS (Version 9.1; SAS, Cary, NC, USA).

Mixed model analyses of variance with within participant factors ‘circadian phase’ (6 levels) and ‘time since start of sleep episode’ (3 levels) (sleep dependent factor) and their interaction were computed for all sleep parameters. Two types of covariance structure were used: unstructured and compound symmetry. Using the same mixed models, we performed analyses in which we controlled the circadian and sleep-dependent effects for further within factors such as brain regions, amplitude threshold or frequency range for SW detection, EEG reference, polarity of the half-waves, and segment (initial versus final) of certain SW parameters (duration and slope). In all these additional analyses we also investigated the interaction between the circadian and sleep dependent factors and the additional control factors to assess whether the circadian and sleep dependent modulation were differentially influenced by these factors. We also controlled for the effect of REM and NREM sleep duration on the studied variables by adding it as continuous measure to the same mixed model. The distribution of residuals was assessed for normality for each single analysis. In order to visualize statistical effect sizes, we calculated the Cohen's *f^2^* effect size (Cohen, 1988):

$$f^{2}=u/v*F\text{,}$$

where *u* and *v* are, respectively, the numerator and denominator degrees of freedom of the F statistic used to determine the corresponding main or interaction effect in the general linear mixed model analysis. In all cases, the averaging of SW parameters within the studied time bins and/or brain regions was weighted for the numbers of detected SWs in that particular time bin or brain region. The target SW measures such as the slope characteristics were highly correlated (Inline Supplementary Table S1) with each other and with the amplitude and the duration. This argues against the application of a conservative correction for multiplicity such as Bonferroni. However, in order to minimize the chance of type I error we set alpha at *P* < 0.005. All statistical analyses were performed in SAS (Version 9.1; SAS, Cary, NC, USA).

Inline Supplementary Table S1

**Table S1:** Representative example of correlations between individual slow measures measured at baseline night (N = 3531 Slow waves, BB0214, Female, 31 years, EEG channel: C3).

Variable	Amplitude	Duration initial segment	Duration final segment	Mean slope initial segment	Mean slope final segment	Maximum slope initial segment	Maximum slope final segment
Amplitude	1	0.16475	0.08064	0.31887	0.3662	0.41186	0.41591
	_	****	****	****	****	****	****
Duration initial segment	0.16475	1	− 0.23783	− 0.86369	0.28204	− 0.42147	0.18372
	****	_	****	****	****	****	****
Duration final segment	0.08064	− 0.23783	1	0.25755	− 0.87838	0.15104	− 0.48273
	****	****	_	****	****	****	****
Mean slope initial segment	0.31887	− 0.86369	0.25755	1	− 0.0698	0.64015	0.0451
	****	****	****	_	***	****	ns
Mean slope final segment	0.3662	0.28204	− 0.87838	− 0.0698	1	0.06831	0.68307
	****	****	****	****	_	***	****
Maximum slope initial segment	0.41186	− 0.42147	0.15104	0.64015	0.06831	1	0.16798
	****	****	****	****	****	_	****
Maximum slope final segment	0.41591	0.18372	− 0.48273	0.0451	0.68307	0.16798	1
	****	****	****	ns	****	****	_

Spearman Rho and significance value are indicated for the studied parameters of all negative half waves of all detected SWs (N = 3531) as measured during the baseline night. (***P < .0005 and ****P < .0001).

Inline Supplementary Table S1

Inline Supplementary Table S1 can be found online at http://dx.doi.org/10.1016/j.neuroimage.2015.05.012.

## Results

### Topography and time course of SW parameters during habitual nocturnal sleep

To validate our SW analysis methodology (Figs. 2A–C), we first analysed the time course and topography of SW parameters during an 8-hour sleep period while participants were sleeping at their habitual nocturnal bedtimes, focusing on the negative half-wave. In accordance with previous studies in humans (Menicucci et al., 2009; Riedner et al., 2007), the incidence, amplitude, duration, and the slope of the negative segment of the SWs, as well as SWA during NREM sleep, showed a strong fronto-posterior gradient (Fig. 2D and Table 1).

**Fig. S2:**
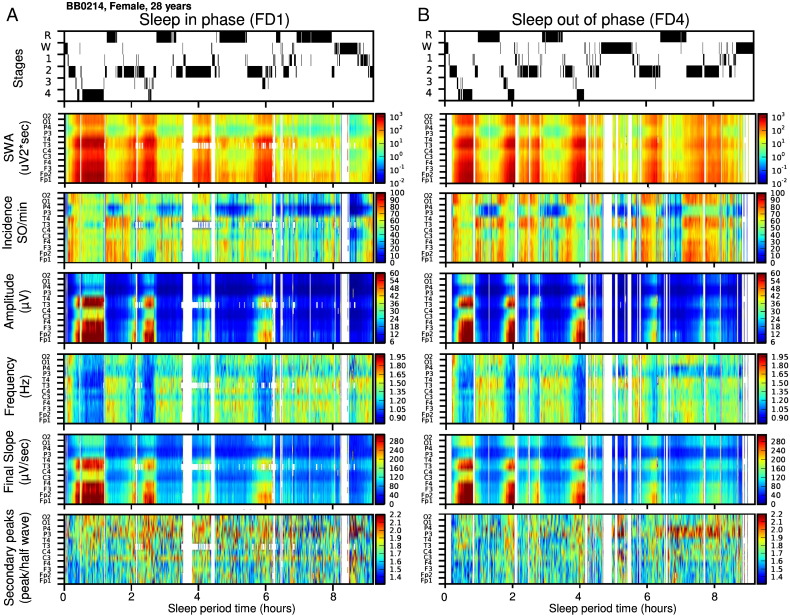
A representative example of the effect of sleep timing on sleep structure and on the time course of multiple slow wave (SW) parameters across each studied EEG derivation (BB0214, Female, 31 years) presented on an interpolated color map. A. Sleep stages during FDn1 when sleep was scheduled to occur at the habitual times, and the interpolated map of corresponding time course (1 min resolution) of slow wave activity (SWA: 0.5–4 Hz), incidence, absolute amplitude, duration, absolute mean slope of the individually detected negative half-waves (> 5 μV, 0.5–4 Hz) of SWs across all studied EEG derivations. B. Sleep stages and the time course of studied SW parameters across all EEG derivations as measured during the 9 hour and 20 minute sleep period FDn4 scheduled 180^o^ (~ 12 h) out of phase with habitual sleep time.

The incidence, amplitude and slope measures were highest over the frontal region and smallest over the posterior region. The duration was shortest over the frontal region and this topographical effect was considerably stronger for the transition period from negative to positive (i.e. duration of the final segment of the negative half-wave) as compared to the transition period from positive to negative polarity of SW half-waves. SW parameters also showed a considerable change in the course of this baseline sleep period. SWA, SW incidence, amplitude, duration, and slope declined throughout the night, and this effect was strongly modulated by brain topography, all in accordance with previous studies (Fig. 2D and Inline Supplementary Fig. S2).

The incidence, amplitude and slope measures were highest over the frontal region and smallest over the posterior region. The duration was shortest over the frontal region and this topographical effect was considerably stronger for the transition period from negative to positive (i.e. duration of the final segment of the negative half-wave) as compared to the transition period from positive to negative polarity of SW half-waves. SW parameters also showed a considerable change in the course of this baseline sleep period. SWA, SW incidence, amplitude, duration, and slope declined throughout the night, and this effect was strongly modulated by brain topography, all in accordance with previous studies (Fig. 2D and Inline Supplementary Fig. S2).

Inline Supplementary Fig. S2

Inline Supplementary Fig. S2 can be found online at http://dx.doi.org/10.1016/j.neuroimage.2015.05.012.

### Circadian and sleep-dependent modulation of sleep efficiency, NREM and REM sleep

**Fig. 3:**
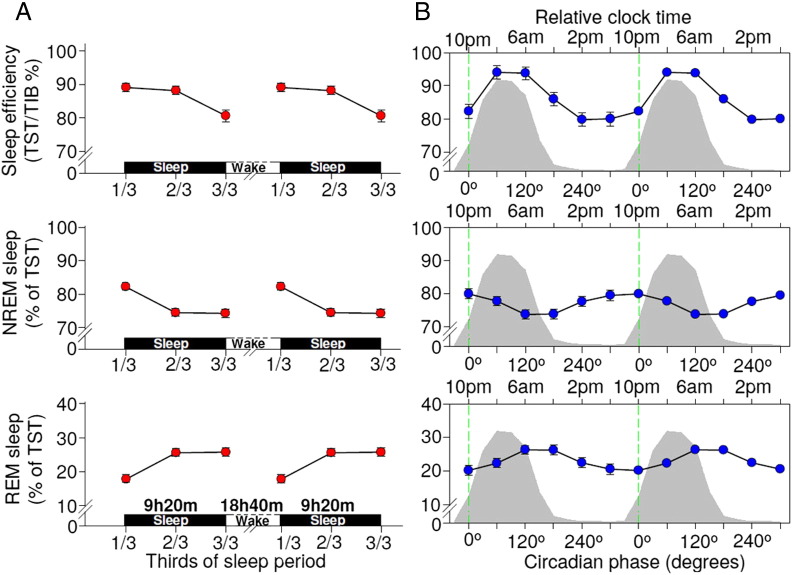
The circadian and sleep-dependent regulation of sleep efficiency, NREM and REM sleep. A. The sleep-dependent regulation of sleep efficiency (*F*_*2*,*220*_ = 12.43, *P* < .0001; *Cohen's f^2^* = 0.11) , NREM sleep (*F*_*2*,*387*_ = 30.12, *P* < .0001; *Cohen's f^2^* = 0.16) and REM sleep (*F*_*2*,*387*_ = 30.12, *P* < .0001; *Cohen's f^2^* = 0.16). Least square mean (Lsmeans) and standard error of the mean (SEM) are presented indicating sleep-dependent estimates at each 3-hour and 6.7-minute intervals (third of the sleep period) measured across all studied circadian phases. Data are double plotted for a better visualization of sleep-dependent rhythmicity. B. The circadian regulation of sleep efficiency (*F*_*5*,*369*_ = 12.07; *P* < 0.001; *Cohen's f^2^* = 0.17), NREM sleep (*F*_*5*,*438*_ = 4.13, *P* = 0.0011; *Cohen's f^2^* = 0.05) and REM sleep (*F*_*5*,*438*_ = 4.13, *P* = 0.0011; *Cohen's f^2^* = 0.05). Lsmeans and SEM indicate circadian phase-dependent estimates at 60^o^ (~ 4 h) bins measured across all studied sleep intervals. Data are double plotted for a better visualization of circadian rhythmicity. Zero phase is set at the dim light melatonin onset indicated by the vertical green dashed line. The grey area in the background represents average melatonin profiles.

To establish whether this implementation of the forced desynchrony protocol confirmed the reported circadian and sleep-dependent regulation of sleep efficiency, REM sleep, and NREM sleep, we computed the circadian and sleep-dependent modulation of these sleep parameters. Mixed model analyses of variance with factors circadian phase and time since start of sleep episode were computed for all sleep parameters. In accordance with previous reports (Dijk and Czeisler, 1995; Wyatt et al., 1999), these sleep parameters displayed the well-known circadian and sleep-dependent variation, with a sleep-dependent decline of sleep efficiency and NREM sleep, a sleep dependent increase in REM sleep, a high sleep efficiency when sleep was scheduled in phase with the melatonin rhythm and a circadian REM sleep peak on the falling limb of the melatonin rhythm (Fig. 3).

### Circadian and sleep-dependent modulation of SW parameters

**Fig. 4:**
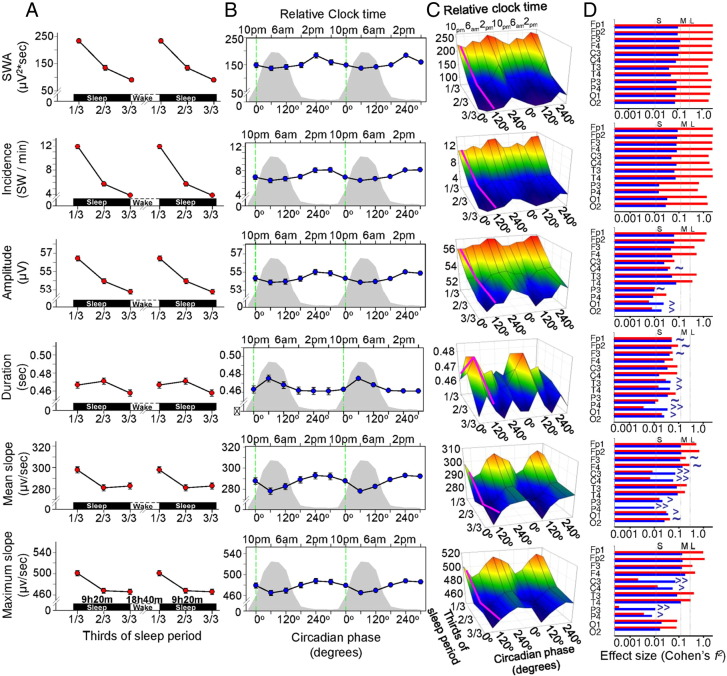
The sleep-dependent and circadian regulation of the slow wave (SW) parameters are presented for the negative half waves. For statistical results of the SW parameters please refer to Table 2. A. The sleep-dependent regulation of SWA (0.5–4 Hz), incidence, absolute amplitude, duration and the absolute slope of the individually detected SWs. Least square mean (Lsmeans) and standard error of the mean (SEM) are presented indicating sleep-dependent estimates at each 3-hour and 6.7-minute intervals (third of the sleep period) measured across all studied circadian phases and EEG derivations. Data are double plotted for a better visualization of sleep-dependent rhythmicity. The circadian regulation of SWA (0.5–4 Hz), incidence, amplitude, period and slope of the individually detected SWs. B. Lsmeans and SEM indicate circadian phase-dependent estimates at 60^o^ (~ 4 h) bins measured across all studied sleep intervals and EEG derivations. Data are double plotted for a better visualization of circadian rhythmicity. Zero phase is set at the dim light melatonin onset indicated by the vertical green line. The grey area in the background represents the average melatonin profile. C. The interaction between the sleep-dependent and circadian regulation of SWs. Lsmeans indicate estimates across 3 sleep intervals (third of the night) and 60^o^ (~ 4 h) circadian bins averaged across all EEG derivations. The oblique purple line indicates the trajectory during baseline night. The 3D representation indicates that for several SW parameters the sleep-dependent and circadian effects interact. D. Effect size (Cohen's *f^2^*) of sleep (red) and circadian phase (blue) dependent regulation of SWA and the studied SW parameters for each studied EEG derivation. The ~ symbol indicates EEG derivation in which circadian effect size is comparable with the sleep-dependent effect size, whereas > symbol indicates EEG derivation in which circadian effect size exceeds the sleep-dependent effect size (> = Circadian *f^2^* > sleep-dependent *f*^2^ up to five times; >> = the Circadian *f^2^* > sleep-dependent *f*^2^ more than five times).

The circadian and sleep-dependent modulation for all SW parameters and SWA in NREM sleep were analysed across all 12 EEG derivations (Fig. 4 and Table 2). The average duration of the analysed NREM per sleep episodes was 361 ± 49 min across all studied nights with an average minimum of 327 ± 29 min at FDn4 and average maximum of 393 ± 59 at Fdn6 during the forced desynchrony protocol.

A prominent and statistically significant sleep-dependent decline was observed for SWA, the incidence, amplitude, duration and slope of the negative segment of the individually detected SWs. All of these parameters, with the exception of the duration parameter, displayed a progressive decline from the first to the final third of the sleep episode (Fig. 4A). We also found a significant effect of circadian phase for all SW parameters (Fig. 4B). The maximum of the circadian rhythms of the incidence, amplitude of SWs, and the mean and maximum slope of the negative half-wave of the SW and SWA were all located in the second half of the biological day and their minima coincided with the biological night, i.e. when melatonin is synthesized (Pevet and Challet, 2011) and core body temperature and brain temperature are low (Dijk and Czeisler, 1995; Landolt et al., 1995). The maximum of the circadian rhythm of the duration of the SW was located in the biological night. Overall the effect sizes of the circadian modulation were smaller than the effects sizes of the sleep-dependent modulation, although for duration and slope parameters the effects size of the circadian modulation was similar to the sleep-dependent effect (Fig. 4D). The duration of the transition period from negative to positive polarity of SW half-waves (i.e. the final segment of the negative half-wave) showed a stronger circadian than sleep-dependent modulation.

For the amplitude, duration, and mean and maximum slope parameters, the interaction of the circadian and sleep-dependent factors was significant (Fig. 4C and Table 2). The interactions imply that the time course of the various SW parameters during sleep depends on the time of day at which sleep is occurring, and this, together with the observed circadian modulation of the studied SW characteristics, demonstrates that the numeric value of these core SW characteristic parameters at the beginning and at the end of a sleep episode depends on when the sleep period was initiated, independent of the duration of wakefulness preceding sleep or the duration of sleep, respectively. All circadian main effects remained significant even after controlling for the duration of NREM sleep at each circadian phase and sleep interval.

Since slow waves are thought to be regulated by the duration of preceding sleep and wakefulness, and sleep duration is modulated by circadian phase, circadian modulation in slow waves could result from carry over effects of variation in sleep duration when the subjects go from sleep episode 1, to 2, etc. In order to investigate the contribution of this potential confound we performed an analysis in which instead of collapsing SW parameters into 6 circadian and 3 sleep dependent time intervals across all sleep episodes, we collapsed the data within the thirds of the night for each individual sleep episode separately. We next included the factors sleep episode (FDn1–FDn7) and thirds of the night in the model. As the sleep episodes were scheduled at different circadian phase (except FDn1 and FDn7) the factor sleep episode represented the circadian and the thirds of the night the sleep dependent component. We ran the analyses while controlling for the sleep dependent and circadian effects for time spent in both NREM and REM sleep during the preceding sleep episode. In this way we eliminated the impact of the partial sleep loss caused by the forced desynchrony protocol on the studied measures. We found highly significant effect of sleep period with medium to large effect size on most SW parameters in addition to a strong sleep dependent effect and a significant interaction between the two factors independently of NREM and REM sleep duration at the preceding sleep episode demonstrating that the circadian phase when sleep is initiated modulates the time course of SW parameters during the night independent of the partial sleep loss present at some circadian phases (Inline Supplementary Table S2).

Since slow waves are thought to be regulated by the duration of preceding sleep and wakefulness, and sleep duration is modulated by circadian phase, circadian modulation in slow waves could result from carry over effects of variation in sleep duration when the subjects go from sleep episode 1, to 2, etc. In order to investigate the contribution of this potential confound we performed an analysis in which instead of collapsing SW parameters into 6 circadian and 3 sleep dependent time intervals across all sleep episodes, we collapsed the data within the thirds of the night for each individual sleep episode separately. We next included the factors sleep episode (FDn1–FDn7) and thirds of the night in the model. As the sleep episodes were scheduled at different circadian phase (except FDn1 and FDn7) the factor sleep episode represented the circadian and the thirds of the night the sleep dependent component. We ran the analyses while controlling for the sleep dependent and circadian effects for time spent in both NREM and REM sleep during the preceding sleep episode. In this way we eliminated the impact of the partial sleep loss caused by the forced desynchrony protocol on the studied measures. We found highly significant effect of sleep period with medium to large effect size on most SW parameters in addition to a strong sleep dependent effect and a significant interaction between the two factors independently of NREM and REM sleep duration at the preceding sleep episode demonstrating that the circadian phase when sleep is initiated modulates the time course of SW parameters during the night independent of the partial sleep loss present at some circadian phases (Inline Supplementary Table S2).

Inline Supplementary Table S2

**Table S2:** Summary of main effects and interactions of factors thirds of the night and sleep episodes scheduled around the circadian clock as well as main effects of covariates REM and NREM duration during the preceding sleep episodes on the studied SW parameters as measured during the forced desynchrony.

SW parameter	Segment	Effect	*DF*	*F* value	*P* value	Cohen's *f^2^*
Incidence		Thirds of the night	2	604.54	< .0001****	23.34^L^
		Sleep episode	6	20.01	< .0001****	0.66^L^
		Sleep episode *Thirds of the night	12	4.86	< .0001****	0.20^M^
		REM duration	1	0.57	ns	
		NREM duration	1	0.86	ns	
Amplitude		Thirds of the night	2	174.61	< .0001****	6.79^L^
		Sleep episode	6	14.34	< .0001****	0.47^L^
		Sleep episode *Thirds of the night	12	3.79	< .0001****	0.16^M^
		REM duration	1	2.76	ns	
		NREM duration	1	1	ns	
Duration	Both	Thirds of the night	2	21.57	< .0001****	0.81^L^
		Sleep episode	6	10.85	< .0001****	0.34^M^
		Sleep episode *Thirds of the night	12	3.63	< .0001****	0.15^M^
		REM duration	1	7.77	0.006	0.04^S^
		NREM duration	1	0.35	ns	
	Initial	Thirds of the night	2	47.75	< .0001****	1.77^L^
		Sleep episode	6	11.33	< .0001****	0.36^L^
		Sleep episode *Thirds of the night	12	4.89	< .0001****	0.20^M^
		REM duration	1	7.54	0.007	0.04^S^
		NREM duration	1	0.38	ns	
	Final	Thirds of the night	2	7.61	0.001*	0.29^L^
		Sleep episode	6	8.52	< .0001****	0.28^M^
		Sleep episode *Thirds of the night	12	2.36	0.007	0.1^S^
		REM duration	1	6.95	0.009	0.04^S^
		NREM duration	1	0.61	ns	
Mean slope	Both	Thirds of the night	2	89.72	< .0001****	3.51^L^
		Sleep episode	6	21.82	< .0001****	0.68^L^
		Sleep episode *Thirds of the night	12	7.14	< .0001****	0.30^M^
		REM duration	1	6.11	0.014	0.03^S^
		NREM duration	1	0.87	ns	
	Initial	Thirds of the night	2	345.05	< .0001****	3.19^L^
		Sleep episode	6	20.94	< .0001****	0.99^L^
		Sleep episode *Thirds of the night	12	4.6	< .0001****	0.17^M^
		REM duration	1	4.01	0.047	0.02^S^
		NREM duration	1	0.38	ns	
	Final	Thirds of the night	2	57.31	< .0001****	2.25^L^
		Sleep episode	6	17.92	< .0001****	0.58^L^
		Sleep episode *Thirds of the night	12	4.84	< .0001****	0.21^M^
		REM duration	1	6.52	0.012	0.03^L^
		NREM duration	1	1.54	ns	
Maximum slope	Both	Thirds of the night	2	120.97	< .0001****	4.78^L^
		Sleep episode	6	22.76	< .0001****	0.71^L^
		Sleep episode *Thirds of the night	12	8.64	< .0001****	0.37^L^
		REM duration	1	4.2	0.042	0.02^L^
		NREM duration	1	0.66	ns	
	Initial	Thirds of the night	2	152.42	< .0001****	5.98^L^
		Sleep episode	6	22.66	< .0001****	0.71^L^
		Sleep episode *Thirds of the night	12	9.69	< .0001****	0.41^L^
		REM duration	1	2.93	ns	
		NREM duration	1	0.11	ns	
	Final	Thirds of the night	2	86.29	< .0001****	3.43^L^
		Sleep episode	6	19.72	< .0001****	0.64^L^
		Sleep episode *Thirds of the night	12	6.03	< .0001****	0.26^M^
		REM duration	1	4.27	0.04	0.02^S^
		NREM duration	1	1.18	ns	

Results for negative half-waves are presented. The Thirds of the night factor includes thirds of the total sleep period (9 h 20 m). The sleep episode factor comprises of 7 sleep episodes scheduled around the circadian clock. REM duration and NREM duration are continuous covariates included in the model and indicate the sleep duration measured always during the previous sleep episode as compared the SW parameters. Segment variable indicates the descending (initial) or the ascending (final) phase of the slow wave (SW) negative half waves. Degree of freedom (DF), *F* values, *P* values, effect size (*Cohen's f^2^*) of main effects, and interactions are indicated for each studied variables as returned from mixed model analyses of variances (*P < .005 and ****P < .0001). Superscripts following effect size values indicate the magnitude of the effects size [small(S): 0.02–0.15, medium (M): 0.15–0.35, large (L): > 0.35]. *P* values and effect sizes for non-significant effects are not indicated. Non-significant trends (< 0.05) are indicated.

Inline Supplementary Table S2

Inline Supplementary Table S2 can be found online at http://dx.doi.org/10.1016/j.neuroimage.2015.05.012.

### Topographical aspects of the circadian and sleep-dependent modulation of SWs

**Fig. 5:**
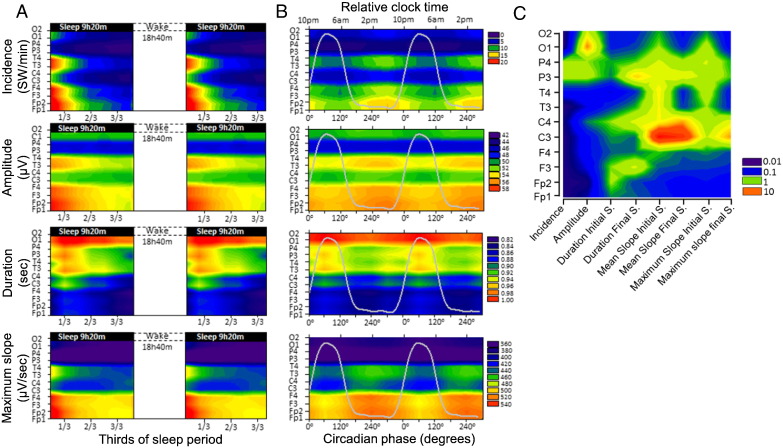
Topographical variation of the sleep dependent and circadian modulation of SW parameters. A. The sleep-dependent regulation of the incidence, amplitude, duration and slope of the individually detected SWs across all studied EEG derivations on an interpolated color map. Data are double plotted for a better visualization of sleep-dependent effects. B. The circadian regulation of the incidence, amplitude, duration and slope of the individually detected SWs across all studied EEG derivations on an interpolated color map. Data are double plotted for a better visualization of circadian rhythmicity. The grey line in the background represents the average melatonin profile. C. The relative magnitude of the sleep dependent and circadian modulation of SW parameters. Values represent ratio of the effects size (Cohen's *f^2^*) between the circadian effects size and the sleep dependent effect size. Warmer colours indicate a dominance of the circadian effect. S. = segment.

We first analysed the circadian and sleep-dependent modulation of the SW parameters across the EEG channels within the frontal, central and posterior brain/cortical regions. Topography had a large main effect as compared to both sleep-dependent and circadian effects, except for incidence, which showed a slightly stronger sleep-dependent modulation (Fig. 4D and Inline Supplementary Tables S3–S4). The effect size of the sleep-dependent and circadian regulation of SW parameters varied across brain regions (Figs. 4D and 5A–C). However, this topographical modulation was greater for the sleep-dependent effect as compared to the circadian. In general, the sleep-dependent effect was greatest over the frontal region and decreased considerably along an antero-posterior gradient, in particular for the amplitude and slope parameters (Figs. 4D and 5A). Although the circadian modulation did not show such a marked topography, for certain brain regions the effect sizes of the circadian modulation exceeded the sleep-dependent modulation (Figs. 4D and 5B). When analysing each EEG derivation separately, the amplitude of the SWs over the occipital region, the duration of the SWs over the temporal and occipital regions and the slope of the SWs over the central, left parietal and occipital regions were predominantly under circadian and not sleep-dependent control. Specifically, the slope over the left and right central brain regions showed a nearly 10-fold greater circadian effect size as compared to the sleep-dependent one.

We first analysed the circadian and sleep-dependent modulation of the SW parameters across the EEG channels within the frontal, central and posterior brain/cortical regions. Topography had a large main effect as compared to both sleep-dependent and circadian effects, except for incidence, which showed a slightly stronger sleep-dependent modulation (Fig. 4D and Inline Supplementary Tables S3–S4). The effect size of the sleep-dependent and circadian regulation of SW parameters varied across brain regions (Figs. 4D and 5A–C). However, this topographical modulation was greater for the sleep-dependent effect as compared to the circadian. In general, the sleep-dependent effect was greatest over the frontal region and decreased considerably along an antero-posterior gradient, in particular for the amplitude and slope parameters (Figs. 4D and 5A). Although the circadian modulation did not show such a marked topography, for certain brain regions the effect sizes of the circadian modulation exceeded the sleep-dependent modulation (Figs. 4D and 5B). When analysing each EEG derivation separately, the amplitude of the SWs over the occipital region, the duration of the SWs over the temporal and occipital regions and the slope of the SWs over the central, left parietal and occipital regions were predominantly under circadian and not sleep-dependent control. Specifically, the slope over the left and right central brain regions showed a nearly 10-fold greater circadian effect size as compared to the sleep-dependent one.

Inline Supplementary Table S3

**Table S3:** Summary of main effects and interactions of the brain topography, sleep dependent and circadian factors on the studied SW parameters as measured during the forced desynchrony.

SW parameter	Segment	Effect	*DF*	*F* value	*P* value	Cohen's *f^2^*
Incidence		Topography	2	697.44	< 0.0001****	6.98^L^
		Sleep dependent	2	728.76	< 0.0001****	7.54^L^
		Circadian	5	35.44	< 0.0001****	0.36^L^
		Topography ∗ Sleep dependent	4	47.46	< 0.0001****	0.93^L^
		Topography ∗ Circadian	10	3.7	< 0.0001****	0.07^S^
		Sleep dependent ∗ Circadian	10	6.81	< 0.0001****	0.08^S^
Amplitude		Topography	2	519.88	< 0.0001****	5.07^L^
		Sleep dependent	2	195.52	< 0.0001****	2.12^L^
		Circadian	5	10.22	< 0.0001****	0.11^S^
		Topography ∗ Sleep dependent	4	33.24	< 0.0001****	0.68^L^
		Topography ∗ Circadian	10	2.01	0.0303	0.04^S^
		Sleep dependent ∗ Circadian	10	3.43	0.0002***	0.04^S^
Duration	Initial	Topography	2	139.29	< 0.0001****	1.35^L^
		Sleep dependent	2	33.82	< 0.0001****	0.36^L^
		Circadian	5	11.61	< 0.0001****	0.12^S^
		Topography ∗ Sleep dependent	4	4.66	0.0013*	0.09^S^
		Topography ∗ Circadian	10	0.25	ns	
		Sleep dependent ∗ Circadian	10	6.46	< 0.0001****	0.08^S^
	Final	Topography	2	845.16	< 0.0001****	8.36^L^
		Sleep dependent	2	52.3	< 0.0001****	0.56^L^
		Circadian	5	20.77	< 0.0001****	0.25^M^
		Topography ∗ Sleep dependent	4	7.86	< 0.0001****	0.16^M^
		Topography ∗ Circadian	10	0.35	ns	
		Sleep dependent ∗ Circadian	10	3.58	0.0001***	0.05^S^
Mean slope	Initial	Topography	2	815.92	< 0.0001****	7.90^L^
		Sleep dependent	2	76.54	< 0.0001****	0.80^L^
		Circadian	5	20.22	< 0.0001****	0.21^M^
		Topography ∗ Sleep dependent	4	26.42	< 0.0001****	0.52^L^
		Topography ∗ Circadian	10	0.97	ns	
		Sleep dependent ∗ Circadian	10	4.06	< 0.0001****	0.05^S^
	Final	Topography	2	1371	< 0.0001****	14.37^L^
		Sleep dependent	2	153.57	< 0.0001****	1.63^L^
		Circadian	5	29.9	< 0.0001****	0.31^M^
		Topography ∗ Sleep dependent	4	10.66	< 0.0001****	0.21^M^
		Topography ∗ Circadian	10	1.68	ns	
		Sleep dependent ∗ Circadian	10	2.2	0.0162	0.03^S^
Maximum slope	Initial	Topography	2	611.73	< 0.0001****	5.88^L^
		Sleep dependent	2	107.72	< 0.0001****	1.13^L^
		Circadian	5	14.03	< 0.0001****	0.14^S^
		Topography ∗ Sleep dependent	4	29.01	< 0.0001****	0.58^L^
		Topography ∗ Circadian	10	1.4	ns	
		Sleep dependent ∗ Circadian	10	3.45	0.0002***	0.04^S^
	Final	Topography	2	1410	< 0.0001****	14.67^L^
		Sleep dependent	2	244.1	< 0.0001****	2.55^L^
		Circadian	5	25.74	< 0.0001****	0.26^M^
		Topography ∗ Sleep dependent	4	13.2	< 0.0001****	0.26^M^
		Topography ∗ Circadian	10	1.77	ns	
		Sleep dependent ∗ Circadian	10	2.6	0.0042*	0.03^S^

Results for negative half-waves are presented. The brain topography factor comprises three main brain regions each including weighted averages over the Frontal (Fp1, Fp2, F3, F4), Central (C3, C4, T3, T4), and Posterior (P3, P4, O1, O2) areas. The sleep-dependent factor includes thirds of the total sleep period (9 h 20 m). The circadian factor comprises of 6 ∗ 60° (~ 4-hourly) bins. The Segment variable indicates the descending (initial) or the ascending (final) phase of the slow wave (SW) negative half waves. Degree of freedom (DF), *F* values, *P* values, effect size (*Cohen's f*^2^) of main effects, and interactions are indicated for each studied variables as returned from mixed model analyses of variances (*P < .005, ***P < .0005, ****P < .0001). Superscripts following effect size values indicate the magnitude of the effects size [small (S): 0.02–0.15, medium (M): 0.15–0.35, large (L): > 0.35]. *P* values and effect sizes for non-significant effects are not indicated. Non-significant trends (< 0.05) are indicated.

Inline Supplementary Table S3

Inline Supplementary Table S4

**Table S4:** Summary of main effects of the sleep dependent and circadian factors on the studied SO parameters within each main brain region as measured during the forced desynchrony.

SW parameters	Segment	Regions	Mean	SD	Sleep dependent effect (H)				Circadian effect (C)			
					*DF*	*F* value	*P* value	Cohen's *f^2^*	*DF*	*F* value	*P* value	Cohen's *f*^2^
Incidence (SO/min)		Frontal	11.62	2.58	2	511.3	< 0.0001****	2.46^L^	5	9.5	< 0.0001****	0.09^S^
		Central	7.28	2.28	2	487.7	< 0.0001****	2.39^L^	5	8.4	< 0.0001****	0.08^S^
		Posterior	3.56	1.88	2	170.4	< 0.0001****	1.22^L^	5	3.1	0.0087	0.03^S^
Amplitude (μV)		Frontal	57.95	2.13	2	300.6	< 0.0001****	1.69^L^	5	8.2	< 0.0001****	0.08^S^
		Central	55.03	2.32	2	170.1	< 0.0001****	0.98^L^	5	5.6	< 0.0001****	0.06^S^
		Posterior	50.92	2.34	2	6.6	0.0015*	0.04^S^	5	0.9	ns	
Duration (s)	Both	Frontal	0.45	0.02	2	20.6	< 0.0001****	0.10^S^	5	6.1	< 0.0001****	0.06^S^
		Central	0.50	0.02	2	13.5	< 0.0001****	0.07^S^	5	6.5	< 0.0001****	0.06^S^
		Posterior	0.52	0.03	2	5.3	0.0053	0.03^S^	5	6.1	< 0.0001****	0.05^S^
	Initial	Frontal	0.23	0.01	2	27.0	< 0.0001****	0.14^S^	5	2.4	0.036	0.02^S^
		Central	0.25	0.01	2	21.5	< 0.0001****	0.11^S^	5	3.2	0.0069	0.03^S^
		Posterior	0.25	0.01	2	24.6	< 0.0001****	0.14^S^	5	4.9	0.0002****	0.05^S^
	Final	Frontal	0.22	0.01	2	13.9	< 0.0001****	0.08^S^	5	8.0	< 0.0001****	0.08^S^
		Central	0.25	0.01	2	4.6	0.0108	0.03^S^	5	6.1	< 0.0001****	0.06^S^
		Posterior	0.27	0.01	2	33.1	< 0.0001****	0.22^M^	5	4.1	0.0012*	0.04^S^
Mean slope (μV)/s	Both	Frontal	318.3	23.72	2	170.9	< 0.0001****	1.02^L^	5	17.1	< 0.0001****	0.16^M^
		Central	271.0	22.53	2	58.9	< 0.0001****	0.33^M^	5	12.4	< 0.0001****	0.11^S^
		Posterior	235.6	24.14	2	9.7	< 0.0001****	0.07^S^	5	2.8	0.0165	0.02^S^
	Initial	Frontal	306.6	22.41	2	134.7	< 0.0001****	0.75^L^	5	10.9	< 0.0001****	0.10^S^
		Central	267.0	21.80	2	28.0	< 0.0001****	0.15^S^	5	6.3	< 0.0001****	0.06^S^
		Posterior	239.2	23.81	2	6.9	0.0011*	0.04^S^	5	3.1	0.0097	0.03^S^
	Final	Frontal	330.0	25.58	2	172.4	< 0.0001****	1.09^L^	5	17.4	< 0.0001****	0.16^S^
		Central	275.0	23.58	2	75.1	< 0.0001****	0.48^L^	5	13.2	< 0.0001****	0.14^S^
		Posterior	232.0	25.28	2	43.8	< 0.0001****	0.35^M^	5	2.6	0.0235	0.02^S^
Max slope (μV)/s	Both	Frontal	527.4	39.50	2	260.7	< 0.0001****	1.54^L^	5	18.6	< 0.0001****	0.16^M^
		Central	464.3	38.47	2	106.7	< 0.0001****	0.63^L^	5	12.0	< 0.0001****	0.10^S^
		Posterior	409.5	42.49	2	14.5	< 0.0001****	0.11^S^	5	1.3	ns	
	Initial	Frontal	530.4	40.66	2	223.0	< 0.0001****	1.25^L^	5	13.3	< 0.0001****	0.12^S^
		Central	473.3	39.94	2	53.9	< 0.0001****	0.31^M^	5	6.8	< 0.0001****	0.06^S^
		Posterior	427.1	45.02	2	3.2	0.0423	0.02^S^	5	1.5	ns	
	Final	Frontal	524.4	38.91	2	254.4	< 0.0001****	1.61^L^	5	18.6	< 0.0001****	0.17^M^
		Central	455.3	37.51	2	150.3	< 0.0001****	0.91^L^	5	15.7	< 0.0001****	0.16^M^
		Posterior	392.0	41.63	2	61.8	< 0.0001****	0.53^L^	5	1.7	ns	

Results for negative half-waves are presented. The main brain regions each included weighted averages over the Frontal (Fp1, Fp2, F3, F4), Central (C3, C4, T3, T4), and Posterior (P3, P4, O1, O2) areas. The sleep-dependent factor comprises thirds of the total sleep period (9 h 20 m). The circadian factor comprised 6 ∗ 60° (~ 4-hourly) bins. The Segment variable indicates the descending (initial) or the ascending (final) phase of the slow wave (SW) negative half waves. When the Segment column indicates ‘Both’ presented values are obtained from the summation (Duration) or the averaging (slope measures) of the values corresponding to the initial and final SW half-wave segments. Absolute mean values, standard deviations, degree of freedom (DF), *F* values, *P* values, effect size (*Cohen's f*^2^) of main effects, and interactions are indicated for each studied variables as returned from mixed model analyses of variances (*P < .005 and ****P < .0001). Superscripts following effect size values indicate the magnitude of the effects size [small (S): 0.02–0.15, medium (M): 0.15–0.35, large (L): > 0.35]. *P* values and effect sizes for non-significant effects are not indicated. Non-significant trends (< 0.05) are indicated.

Inline Supplementary Table S4

Inline Supplementary Tables S3 and S4 can be found online at http://dx.doi.org/10.1016/j.neuroimage.2015.05.012.

### The effect of the amplitude threshold and frequency range used for SW detection, EEG reference, SW half wave polarity, and direction of transition between polarities on the circadian and sleep-dependent modulation of the SW parameters

We analysed the extent to which the circadian and sleep-dependent regulation of SW was dependent on some of the rather arbitrary selection criteria such as the amplitude threshold used for SW detection, studied frequency range, EEG reference, the polarity of the SW half waves, and the direction of transition between polarities (initial versus final segment), because these parameters have been reported to influence the observed sleep-dependent modulation of SWs (Bersagliere and Achermann, 2010). We performed these analyses across all EEG derivations.

We first ran the mixed model analyses of variance adding the factor ‘amplitude threshold’ for SW detection in addition to the circadian and sleep-dependent factors. For this we analysed the SW parameters calculated for the 5 and 37.5 μV threshold, which allowed a comparison of all existing SWs with the actual high amplitude SWs considered in most studies. The analysis was performed for the negative half-waves. As expected, we found that the ‘amplitude threshold’ factor had a large main effect on all studied SW parameters (Inline Supplementary Table S5). However, the reported circadian and sleep-dependent effects remained highly significant (Inline Supplementary Table S5). The amplitude threshold also strongly modulated the sleep-dependent regulation of all SW parameters and the circadian modulation of the amplitude, incidence and duration parameters. Interestingly, the circadian modulation of the slope measures was not dependent on the amplitude threshold (Inline Supplementary Table S5).

We first ran the mixed model analyses of variance adding the factor ‘amplitude threshold’ for SW detection in addition to the circadian and sleep-dependent factors. For this we analysed the SW parameters calculated for the 5 and 37.5 μV threshold, which allowed a comparison of all existing SWs with the actual high amplitude SWs considered in most studies. The analysis was performed for the negative half-waves. As expected, we found that the ‘amplitude threshold’ factor had a large main effect on all studied SW parameters (Inline Supplementary Table S5). However, the reported circadian and sleep-dependent effects remained highly significant (Inline Supplementary Table S5). The amplitude threshold also strongly modulated the sleep-dependent regulation of all SW parameters and the circadian modulation of the amplitude, incidence and duration parameters. Interestingly, the circadian modulation of the slope measures was not dependent on the amplitude threshold (Inline Supplementary Table S5).

Inline Supplementary Table S5

**Table S5:** Summary of main effects and interactions of SW detection amplitude threshold, sleep dependent and circadian factors on the studied SW parameters as measured during the forced desynchrony.

SW parameter	Segment	Effect	DF	F value	P value	Cohen's *f ^2^*
Incidence		Amplitude threshold	1	27,334.6	< 0.0001****	206.87^L^
		Sleep dependent	2	49.88	< 0.0001****	0.87^L^
		Circadian	5	11.28	< 0.0001****	0.18^M^
		Amplitude threshold ∗ Circadian	5	4.07	0.0013*	0.06^S^
		Amplitude threshold ∗ Sleep dependent	2	81.61	< 0.0001****	1.42^L^
		Sleep dependent ∗ Circadian	10	1.77	ns	
Amplitude		Amplitude threshold	1	42,229.4	< 0.0001****	336.91^L^
		Sleep dependent	2	624.93	< 0.0001****	10.62^L^
		Circadian	5	27.95	< 0.0001****	0.44^L^
		Amplitude threshold ∗ Circadian	5	2.47	0.0324	0.04^S^
		Amplitude threshold ∗ Sleep dependent	2	105.44	< 0.0001****	1.79^L^
		Sleep dependent ∗ Circadian	10	4.78	< 0.0001****	0.09^S^
Duration	Initial	Amplitude threshold	1	5360.31	< 0.0001****	40.63^L^
		Sleep dependent	2	50.2	< 0.0001****	0.83^L^
		Circadian	5	5.52	< 0.0001****	0.09^S^
		Amplitude threshold ∗ Circadian	5	5.14	0.0002***	0.08^S^
		Amplitude threshold ∗ Sleep dependent	2	12.8	< 0.0001****	0.21^M^
		Sleep dependent ∗ Circadian	10	5.83	< 0.0001****	0.11^S^
	Final	Amplitude threshold	1	3897.31	< 0.0001****	30.36^L^
		Sleep dependent	2	12.28	< 0.0001****	0.21^M^
		Circadian	5	9.69	< 0.0001****	0.17^M^
		Amplitude threshold ∗ Circadian	5	6.55	< 0.0001****	0.10^S^
		Amplitude threshold ∗ Sleep dependent	2	29.64	< 0.0001****	0.50^L^
		Sleep dependent ∗ Circadian	10	4.16	< 0.0001****	0.08^S^
Mean slope	Initial	Amplitude threshold	1	13,508.1	< 0.0001****	101.74^L^
		Sleep dependent	2	335.98	< 0.0001****	5.65^L^
		Circadian	5	36.78	< 0.0001****	0.59^L^
		Amplitude threshold ∗ Circadian	5	0.58	ns	
		Amplitude threshold ∗ Sleep dependent	2	74.95	< 0.0001****	1.26^L^
		Sleep dependent ∗ Circadian	10	2.33	0.011	0.04^S^
	Final	Amplitude threshold	1	8979.5	< 0.0001****	73.57^L^
		Sleep dependent	2	330.59	< 0.0001****	5.77^L^
		Circadian	5	42.85	< 0.0001****	0.76^L^
		Amplitude threshold ∗ Circadian	5	1.13	ns	
		Amplitude threshold ∗ Sleep dependent	2	43.05	< 0.0001****	0.75^L^
		Sleep dependent ∗ Circadian	10	1.58	ns	
Maximum slope	Initial	Amplitude threshold	1	13,469.6	< 0.0001****	101.55^L^
		Sleep dependent	2	402.52	< 0.0001****	6.77^L^
		Circadian	5	33.97	< 0.0001****	0.53^L^
		Amplitude threshold ∗ Circadian	5	0.42	ns	
		Amplitude threshold ∗ Sleep dependent	2	60.43	< 0.0001****	1.02^L^
		Sleep dependent ∗ Circadian	10	3.09	0.0008**	0.06^S^
	Final	Amplitude threshold	1	12,797.8	< 0.0001****	105.06^L^
		Sleep dependent	2	470.75	< 0.0001****	7.98^L^
		Circadian	5	45.73	< 0.0001****	0.75^L^
		Amplitude threshold ∗ Circadian	5	0.79	ns	
		Amplitude threshold ∗ Sleep dependent	2	39.04	< 0.0001****	0.66^L^
		Sleep dependent ∗ Circadian	10	2.75	0.003*	0.05^S^

Results for negative half-waves are presented. The amplitude threshold factor comprises the <− 5 μV and the <− 37.5 μV detection thresholds. The sleep-dependent factor includes thirds of the total sleep period (9 h 20 m). The circadian factor comprises 6 ∗ 60° bins. The Segment variable indicates the descending (initial) or the ascending (final) phase of the slow wave (SW) negative half waves. Degree of freedom (DF), *F* values, *P* values, effect size (*Cohen's f*^2^) of main effects, and interactions are indicated for each studied variables as returned from mixed model analyses of variances (*P < .005, **P < .001, ***P < .0005, ****P < .0001). Superscripts following effect size values indicate the magnitude of the effects size [small (S): 0.02–0.15, medium (M): 0.15–0.35, large (L): > 0.35]. *P* values and effect sizes for non-significant effects are not indicated. Non-significant trends (< 0.05) are indicated.

Inline Supplementary Table S5

Inline Supplementary Table S5 can be found online at http://dx.doi.org/10.1016/j.neuroimage.2015.05.012.

**Fig. S3:**
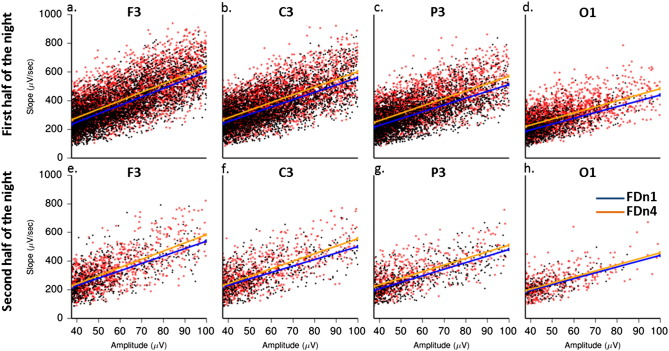
A representative example of the effect of sleep timing on the association between the absolute mean slope and the amplitude of the SWs during the first and second half of the sleep episode (BB0135, Female, 23 years). Fitted least square mean regression lines between the absolute mean slope and the amplitude of the SWs (negative half waves > 37.5 μV, absolute value) as measured during the first and the second half of the night when sleeping at habitual nocturnal bedtimes s (FDn1) and the sleep episode starting 12 h later, i.e. during the biological day (FDn4). The data show that when sleeping during the biological day, for any given amplitude the slope is greater, than when sleeping during the biological night.

Finally we investigated the independence of the circadian variation of the SW slope measures from the circadian variation of SW amplitude. In other words, we investigated whether the circadian variation in slope was driven primarily by a circadian variation in the amplitude of the slow waves. First, we re-run the above analyses adding the SW amplitude values in the model as a continuous covariate in addition to the sleep-dependent and circadian factors and we found that the circadian modulation of both mean slope (F_5,530_ = 23.25; *P* < 0.0001, Cohen's *f*^2^ = 0.22) and maximum slope (F_5,520_ = 21.83; *P* < 0.0001, Cohen's *f*^2^ = 0.21) of the SWs remained highly significant. Second, we calculated the intercept and the slope of a linear regression model fitted using the least squares approach between the mean slope and the amplitude of each individually detected slow wave in each EEG derivation separately for the first and the second half of the night as measured during the baseline sleep episode (FDn1) and the sleep episode scheduled 12 h out of phase (FDn4) (Inline Supplementary Fig. S3). The correlations included on average 2650 in the first and 813 individual SWs in the second half of the night for each participant and EEG channel. Only SWs over 37.5 μV were included in the analyses (Inline Supplementary Fig. S3). We then statistically compared the intercept and the slope of the regression line between the FDn1 and FDn4 and between the first and second half of the sleep episodes after collapsing the data across all studied EEG derivations. We found that the intercept was significantly (F_1,30.3_ = 15.95; P = 0.0004, Cohen's *f^2^* = 0.53) larger for FDn4 [Estimate(95% CI): 48.8(43.6–54.7)] compared to FDn1 [Estimate(95% CI): 43.1(38.4–48.2)] independently of half of the night. Similarly, the slope of the regression line was significantly (F_1,31.2_ = 29.39; *P* < 0.0001, Cohen's *f*^2^ = 0.96) steeper for FDn4 [Estimate (95% CI): 4.5(4.3–5.6)] compared to FDn1 [Estimate (95% CI): 4.3(4.2–4.4)]. Thus circadian phase modulates the slope of SWs, independent from the circadian variation in amplitude.

Finally we investigated the independence of the circadian variation of the SW slope measures from the circadian variation of SW amplitude. In other words, we investigated whether the circadian variation in slope was driven primarily by a circadian variation in the amplitude of the slow waves. First, we re-run the above analyses adding the SW amplitude values in the model as a continuous covariate in addition to the sleep-dependent and circadian factors and we found that the circadian modulation of both mean slope (F_5,530_ = 23.25; *P* < 0.0001, Cohen's *f*^2^ = 0.22) and maximum slope (F_5,520_ = 21.83; *P* < 0.0001, Cohen's *f*^2^ = 0.21) of the SWs remained highly significant. Second, we calculated the intercept and the slope of a linear regression model fitted using the least squares approach between the mean slope and the amplitude of each individually detected slow wave in each EEG derivation separately for the first and the second half of the night as measured during the baseline sleep episode (FDn1) and the sleep episode scheduled 12 h out of phase (FDn4) (Inline Supplementary Fig. S3). The correlations included on average 2650 in the first and 813 individual SWs in the second half of the night for each participant and EEG channel. Only SWs over 37.5 μV were included in the analyses (Inline Supplementary Fig. S3). We then statistically compared the intercept and the slope of the regression line between the FDn1 and FDn4 and between the first and second half of the sleep episodes after collapsing the data across all studied EEG derivations. We found that the intercept was significantly (F_1,30.3_ = 15.95; P = 0.0004, Cohen's *f^2^* = 0.53) larger for FDn4 [Estimate(95% CI): 48.8(43.6–54.7)] compared to FDn1 [Estimate(95% CI): 43.1(38.4–48.2)] independently of half of the night. Similarly, the slope of the regression line was significantly (F_1,31.2_ = 29.39; *P* < 0.0001, Cohen's *f*^2^ = 0.96) steeper for FDn4 [Estimate (95% CI): 4.5(4.3–5.6)] compared to FDn1 [Estimate (95% CI): 4.3(4.2–4.4)]. Thus circadian phase modulates the slope of SWs, independent from the circadian variation in amplitude.

Inline Supplementary Fig. S3

Inline Supplementary Fig. S3 can be found online at http://dx.doi.org/10.1016/j.neuroimage.2015.05.012.

**Fig. S4:**
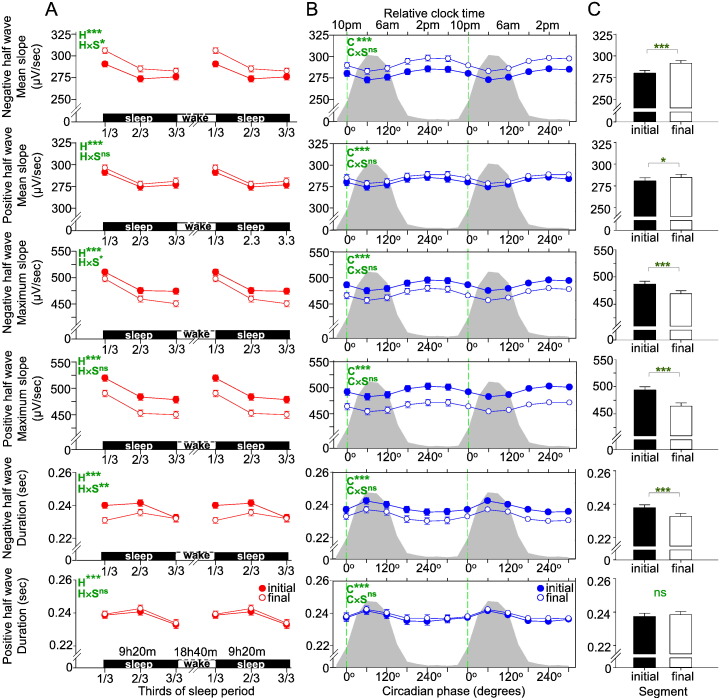
The effect of SW half-wave segment on the duration and slope measures of both positive and negative SW measures. A. The effect of half-wave segment (initial versus final) and sleep-dependent regulation on the absolute slope and duration of SW for both negative and positive half-waves. Least square mean (Lsmeans) and standard error of the mean (SEM) are presented indicating sleep-dependent estimates at each 3-hour and 6.7-minute intervals (third of the sleep period) measured across all studied circadian phases and EEG derivations for both the initial and final segments (open circles = initial segment; filled bars = final segment). Data are double plotted for a better visualization of sleep-dependent rhythmicity. The level of significance is indicated for the main effect of sleep-dependent (H) regulation and the interaction (H ∗ S) with the segment of the SW half-wave factor (*P < .005, **P < .001, ***P < .0005). B. The circadian regulation of SW half-wave duration and various types of slope measures (absolute values) as a function of half-wave polarity and half-wave segment. Lsmeans and SEM indicate circadian phase-dependent estimates at 60^o^ (~ 4 h) bins measured across all studied sleep intervals and EEG derivations for both the initial and final segments (open circles = initial segment; filled bars = final segment). Data are double plotted for a better visualization of circadian rhythmicity. Zero phase is set at the dim light melatonin onset. The level of significance is indicated for the main effect of circadian regulation (C) and the interaction (C ⁎ S) with the segment of the SW half-wave factor (***P < .0005). C. The main effect of the segment of the half-wave, independent of the sleep-dependent and circadian factors on the slope measures (absolute values) and duration of both positive and negative SW half-waves (open bars = initial segment; filled bars = final segment) (*P < .005, ***P < .0005).

We performed additional analyses to assess the robustness of the observed circadian variation by investigating the effects of frequency band width (Inline Supplementary Table S6), the effects of EEG reference (comparing the Pz common reference versus the contralateral mastoid reference: A1 and A2) (Inline Supplementary Table S7), the effects of polarity of SW half waves (Inline Supplementary Table S8) and the effect of ascending versus descending transitions between the positive and negative half-waves (initial versus final segment) (Inline Supplementary Table S9, Inline Supplementary Fig. S4). We found that the circadian modulation of SW parameters was independent whereas the sleep dependent modulation of SW characteristics was modulated by some of these factors.

We performed additional analyses to assess the robustness of the observed circadian variation by investigating the effects of frequency band width (Inline Supplementary Table S6), the effects of EEG reference (comparing the Pz common reference versus the contralateral mastoid reference: A1 and A2) (Inline Supplementary Table S7), the effects of polarity of SW half waves (Inline Supplementary Table S8) and the effect of ascending versus descending transitions between the positive and negative half-waves (initial versus final segment) (Inline Supplementary Table S9, Inline Supplementary Fig. S4). We found that the circadian modulation of SW parameters was independent whereas the sleep dependent modulation of SW characteristics was modulated by some of these factors.

Inline Supplementary Fig. S4

Inline Supplementary Table S6

**Table S6:** Summary of main effects and interactions of the frequency range, sleep dependent and circadian factors on the studied SW parameters as measured during the forced desynchrony.

SW parameter	Segment	Effect	*DF*	*F* value	*P* value	Cohen's *f*^2^
Incidence		Frequency range	1	22.3	< 0.0001****	0.25^M^
		Sleep dependent	2	1119.14	< 0.0001****	18.45^L^
		Circadian	5	43	< 0.0001****	0.63^L^
		Frequency range ∗ Circadian	5	0.16	ns	
		Frequency range ∗ Sleep dependent	2	2.41	ns	
		Sleep dependent ∗ Circadian	10	7.08	< 0.0001****	0.15^S^
Amplitude		Frequency range	1	17.16	< 0.0001****	0.18^M^
		Sleep dependent	2	553.75	< 0.0001****	9.27^L^
		Circadian	5	32.64	< 0.0001****	0.59^L^
		Frequency range ∗ Circadian	5	0.07	ns	
		Frequency range ∗ Sleep dependent	2	0.37	ns	
		Sleep dependent ∗ Circadian	10	5.04	< 0.0001****	0.11^S^
Duration	Initial	Frequency range	1	186	< 0.0001****	1.63^L^
		Sleep dependent	2	45.6	< 0.0001****	0.68^L^
		Circadian	5	9.48	< 0.0001****	0.15^S^
		Frequency range ∗ Circadian	5	0.28	ns	
		Frequency range ∗ Sleep dependent	2	0.64	ns	
		Sleep dependent ∗ Circadian	10	5.46	< 0.0001****	0.11^S^
	Final	Frequency range	1	118.15	< 0.0001****	1.13^L^
		Sleep dependent	2	12.52	< 0.0001****	0.21^M^
		Circadian	5	20.02	< 0.0001****	0.39^L^
		Frequency range ∗ Circadian	5	0.34	ns	
		Frequency range ∗ Sleep dependent	2	0.27	ns	
		Sleep dependent ∗ Circadian	10	3.77	< 0.0001****	0.08^S^
Mean slope	Initial	Frequency range	1	147.19	< 0.0001****	1.48^L^
		Sleep dependent	2	165.91	< 0.0001****	2.61^L^
		Circadian	5	40.7	< 0.0001****	0.63^L^
		Frequency range ∗ Circadian	5	0.37	ns	
		Frequency range ∗ Sleep dependent	2	0.43	ns	
		Sleep dependent ∗ Circadian	10	3.5	0.0002***	0.07^S^
	Final	Frequency range	1	82.28	< 0.0001****	0.87^L^
		Sleep dependent	2	185.93	< 0.0001****	3.38^L^
		Circadian	5	46.71	< 0.0001****	1.03^L^
		Frequency range ∗ Circadian	5	0.5	ns	
		Frequency range ∗ Sleep dependent	2	0.14	ns	
		Sleep dependent ∗ Circadian	10	2.24	0.015	0.05^S^
Maximum slope	Initial	Frequency range	1	71.03	< 0.0001****	0.77^L^
		Sleep dependent	2	284.52	< 0.0001****	4.45^L^
		Circadian	5	38.03	< 0.0001****	0.55^L^
		Frequency range ∗ Circadian	5	0.11	ns	
		Frequency range ∗ Sleep dependent	2	0.12	ns	
		Sleep dependent ∗ Circadian	10	3.53	0.0002***	0.07^S^
	Final	Frequency range	1	56.81	< 0.0001****	0.62^L^
		Sleep dependent	2	381.97	< 0.0001****	6.58^L^
		Circadian	5	57.46	< 0.0001****	1.07^L^
		Frequency range ∗ Circadian	5	0.2	ns	
		Frequency range ∗ Sleep dependent	2	0.09	ns	
		Sleep dependent ∗ Circadian	10	2.56	0.0051	0.06^S^

We assessed the effect of the frequency band-width used for SW detection. All SW parameters for the frequency band 0.5–2 Hz and 0.5–4 Hz were analysed in the same mixed model adding the factor ‘frequency range’. We found this factor to have a significant effect on most SW parameters, however, the sleep-dependent and circadian main effects remained significant and were not modulated by the frequency range used for the analysis. We assessed the effect of the frequency band-width used for SW detection. All SW parameters for the frequency band 0.5–2 Hz and 0.5–4 Hz were analysed in the same mixed model adding the factor ‘frequency range’. We found this factor to have a significant effect on most SW parameters, however, the sleep-dependent and circadian main effects remained significant and were not modulated by the frequency range used for the analysis. Results for negative half-waves are presented. The frequency range factor comprises the 0.5–2 Hz and the 0.5–4 Hz band-width. The sleep-dependent factor includes thirds of the total sleep period (9 h 20 m). The circadian factor comprises 6 ∗ 60° bins. The Segment variable indicates the descending (initial) or the ascending (final) phase of the slow wave (SW) negative half waves. Degree of freedom (DF), *F* values, *P* values, effect size (*Cohen's f*^2^) of main effects, and interactions are indicated for each studied variables as returned from mixed model analyses of variances (***P < .0005 and ****P < .0001). Superscripts following effect size values indicate the magnitude of the effects size [small(S): 0.02–0.15, medium (M): 0.15–0.35, large (L): > 0.35]. *P* values and effect sizes for non-significant effects are not indicated. Non-significant trends (< 0.05) are indicated.

Inline Supplementary Table S6

Inline Supplementary Table S7

**Table S7:** Summary of main effects and interactions of EEG reference, sleep dependent and circadian factors on the studied SW parameters as measured during the forced desynchrony.

SW parameter	Segment	Effect	*DF*	*F* value	*P* value	Cohen's *f*^2^
Incidence		EEG reference	1	313.98	< 0.0001****	3.19^L^
		Sleep dependent	2	1094.29	< 0.0001****	17.78^L^
		Circadian	5	45.47	< 0.0001****	0.67^L^
		EEG reference ∗ Sleep dependent	2	24.31	< 0.0001****	0.40^L^
		EEG reference ∗ Circadian	5	1.6	ns	0.02^S^
		Sleep dependent ∗ Circadian	10	6.19	< 0.0001****	0.13^S^
Amplitude		EEG reference	1	89.49	< 0.0001****	0.75^L^
		Sleep dependent	2	352.32	< 0.0001****	5.86^L^
		Circadian	5	33.41	< 0.0001****	0.58^L^
		EEG reference ∗ Sleep dependent	2	0.22	ns	
		EEG reference ∗ Circadian	5	0.74	ns	
		Sleep dependent ∗ Circadian	10	4.92	< 0.0001****	0.10^S^
Duration	Initial	EEG reference	1	1.65	ns	
		Sleep dependent	2	25.57	< 0.0001****	0.42^L^
		Circadian	5	17.78	< 0.0001****	0.28^M^
		EEG reference ∗ Sleep dependent	2	17.81	< 0.0001****	0.29^M^
		EEG reference ∗ Circadian	5	1.99	ns	
		Sleep dependent ∗ Circadian	10	4.87	< 0.0001****	0.09^S^
	Final	EEG reference	1	362.36	< 0.0001****	2.94^L^
		Sleep dependent	2	10.77	< 0.0001****	0.18^M^
		Circadian	5	22.95	< 0.0001****	0.39^L^
		EEG reference ∗ Sleep dependent	2	1.47	ns	
		EEG reference ∗ Circadian	5	0.22	ns	
		Sleep dependent ∗ Circadian	10	4.2	< 0.0001****	0.08^S^
Mean slope	Initial	EEG reference	1	54.12	< 0.0001****	0.47^L^
		Sleep dependent	2	175.86	< 0.0001****	2.86^L^
		Circadian	5	61.83	< 0.0001****	0.95^L^
		EEG reference ∗ Sleep dependent	2	7.37	0.0009**	0.12^S^
		EEG reference ∗ Circadian	5	1.79	ns	
		Sleep dependent ∗ Circadian	10	3.45	0.0002***	0.07^S^
	Final	EEG reference	1	320.74	< .0001****	2.89^L^
		Sleep dependent	2	116.82	< .0001****	2.00^L^
		Circadian	5	56.63	< .0001****	1.08^L^
		EEG reference ∗ Sleep dependent	2	0.96	ns	
		EEG reference ∗ Circadian	5	0.94	ns	
		Sleep dependent ∗ Circadian	10	2.84	0.0019*	0.06^S^
Maximum slope	Initial	EEG reference	1	6.56	0.0117	0.06^S^
		Sleep dependent	2	236.03	< 0.0001****	3.82^L^
		Circadian	5	57.1	< 0.0001****	0.85^L^
		EEG reference ∗ Sleep dependent	2	3.34	0.0388	0.05^S^
		EEG reference ∗ Circadian	5	0.99	ns	
		Sleep dependent ∗ Circadian	10	3.41	0.0002***	0.07^S^
	Final	EEG reference	1	201.94	< 0.0001****	1.85^L^
		Sleep dependent	2	219.44	< 0.0001****	3.69^L^
		Circadian	5	67.17	< 0.0001****	1.18^L^
		EEG reference ∗ Sleep dependent	2	2.51	ns	
		EEG reference ∗ Circadian	5	0.8	ns	
		Sleep dependent ∗ Circadian	10	2.38	0.0092	0.05^S^

We investigated the influence of the EEG reference (common reference versus contralateral mastoid reference) used for SW analyses on the sleep dependent and circadian variation of SW parameters. The factor ‘EEG reference’ had a significant main effect on most studied SW parameters except the duration of the initial segment of SW half waves independent of the sleep dependent and circadian factors which remained strongly significant. Whereas the EEG reference significantly modulated the sleep dependent variation of some SW parameters such as the incidence, amplitude, as well as the duration, and slope of the initial segments of the half waves, the circadian regulation of SWs was not modulated by the EEG reference. Results for negative half-waves are presented. The EEG reference factor comprises the common reference (where all electrodes are referenced to Pz) and the contralateral mastoid reference (where all EEG electrodes from a certain brain hemisphere are referenced to the mastoid area of the opposite hemisphere: A1 and A2). The sleep-dependent factor includes thirds of the total sleep period (9 h 20 m). The circadian factor comprises 6 ∗ 60° bins. The Segment variable indicates the descending (initial) or the ascending (final) phase of the slow wave (SW) negative half waves. Degree of freedom (DF), *F* values, *P* values, effect size (*Cohen's f*^2^) of main effects, and interactions are indicated for each studied variables as returned from mixed model analyses of variances (*P < .005, **P < .001, ***P < .0005, ****P < .0001). Superscripts following effect size values indicate the magnitude of the effects size [small (S): 0.02–0.15, medium (M): 0.15–0.35, large (L): > 0.35]. *P* values and effect sizes for non-significant effects are not indicated. Non-significant trends (< 0.05) are indicated.

Inline Supplementary Table S7

Inline Supplementary Table S8

**Table S8:** Summary of main effects and interactions of SW half wave polarity, sleep dependent and circadian factors on the studied SW parameters as measured during the forced desynchrony.

SW parameter	Segment	Effect	*DF*	*F* value	*P* value	Cohen's *f*^2^
Incidence		Polarity	1	0.04	ns	
		Sleep dependent	2	1024.41	< 0.0001****	17.03^L^
		Circadian	5	44.51	< 0.0001****	0.65^L^
		Polarity ∗ Circadian	5	0.01	ns	
		Polarity ∗ Sleep dependent	2	0.07	ns	
		Sleep dependent ∗ Circadian	10	7.34	< 0.0001****	0.16^M^
Amplitude		Polarity	1	8.64	0.004*	0.08^S^
		Sleep dependent	2	440.31	< 0.0001****	7.19^L^
		Circadian	5	23.43	< 0.0001****	0.41^L^
		Polarity ∗ Circadian	5	0.08	ns	
		Polarity ∗ Sleep dependent	2	0.08	ns	
		Sleep dependent ∗ Circadian	10	4.6	< 0.0001****	0.10^S^
Duration	Initial	Polarity	1	0.47	ns	
		Sleep dependent	2	32.11	< 0.0001****	0.50^L^
		Circadian	5	11.3	< 0.0001****	0.18^M^
		Polarity ∗ Circadian	5	0.18	ns	
		Polarity ∗ Sleep dependent	2	0.34	ns	
		Sleep dependent ∗ Circadian	10	4.13	< 0.0001****	0.08^S^
	Final	Polarity	1	21.2	< 0.0001****	0.16^M^
		Sleep dependent	2	11.84	< 0.0001****	0.20^M^
		Circadian	5	8.97	< 0.0001****	0.15^S^
		Polarity ∗ Circadian	5	0.19	ns	
		Polarity ∗ Sleep dependent	2	2.92	ns	
		Sleep dependent ∗ Circadian	10	3.69	< 0.0001****	0.07^S^
Mean slope	Initial	Polarity	1	0.47	ns	
		Sleep dependent	2	123.33	< 0.0001****	1.94^L^
		Circadian	5	35.57	< 0.0001****	0.54^L^
		Polarity ∗ Circadian	5	0.2	ns	
		Polarity ∗ Sleep dependent	2	0.03	ns	
		Sleep dependent ∗ Circadian	10	3.76	< 0.0001****	0.07^S^
	Final	Polarity	1	14.22	0.0003***	0.12^S^
		Sleep dependent	2	94.34	< 0.0001****	1.59^L^
		Circadian	5	22.65	< 0.0001****	0.41^L^
		Polarity ∗ Circadian	5	0.57	ns	
		Polarity ∗ Sleep dependent	2	2.68	ns	
		Sleep dependent ∗ Circadian	10	2.65	0.0037*	0.05^S^
Maximum slope	Initial	Polarity	1	12.6	0.0006**	0.12^S^
		Sleep dependent	2	197.96	< 0.0001****	3.16^L^
		Circadian	5	40.39	< 0.0001****	0.61^L^
		Polarity ∗ Circadian	5	0.22	ns	
		Polarity ∗ Sleep dependent	2	0.55	ns	
		Sleep dependent ∗ Circadian	10	3.58	0.0001****	0.07^S^
	Final	Polarity	1	5.9	0.0168	0.05^S^
		Sleep dependent	2	252.94	< 0.0001****	4.14^L^
		Circadian	5	33.58	< 0.0001****	0.58^L^
		Polarity ∗ Circadian	5	0.56	ns	
		Polarity ∗ Sleep dependent	2	1.26	ns	
		Sleep dependent ∗ Circadian	10	2.67	0.0035*	0.05^S^

We assessed the effect of polarity on the studied SW parameters in addition to the circadian and sleep-dependent factors. We found that factor ‘polarity’ had an independent significant effect on most SW parameters except incidence, duration and mean slope of the initial segment and the maximum slope of the final segment of SW half-waves. However, overall the effect size of polarity was smaller as compared to the sleep-dependent and the circadian regulation, which yielded strong significance and no significant interaction with polarity. The polarity factor includes the SW parameters for the negative and positive half-waves. The sleep-dependent factor includes thirds of the total sleep period (9 h 20 m). The circadian factor comprised 6 ∗ 60° bins. The Segment variable indicates the descending (initial) or the ascending (final) phase of the slow wave (SW) negative half waves. Degree of freedom (DF), *F* values, *P* values, effect size (*Cohen's f*^2^) of main effects, and interactions are indicated for each studied variables as returned from mixed model analyses of variances (*P < .005, **P < .001, ***P < .0005, ****P < .0001). Superscripts following effect size values indicate the magnitude of the effects size [small (S): 0.02–0.15, medium (M): 0.15–0.35, large (L): > 0.35]. *P* values and effect sizes for non-significant effects are not indicated. Non-significant trends (< 0.05) are indicated.

Inline Supplementary Table S8

Inline Supplementary Table S9

**Table S9:** Summary of main effects and interactions of the SW half wave segment, sleep dependent and circadian factors on the studied SW parameters as measured during the forced desynchrony.

Polarity	SW parameter	Effect	*DF*	*F* value	*P* value	Cohen's *f ^2^*
Negative	Duration	Segment	1	31.7	< 0.0001****	0.26^M^
		Sleep dependent	2	14.8	< 0.0001****	0.24^M^
		Circadian	5	14.63	< 0.0001****	0.25^M^
		Segment ∗ Circadian	5	0.26	ns	
		Segment ∗ Sleep dependent	2	9.41	0.0002***	0.15^S^
		Sleep dependent ∗ Circadian	10	4.07	< 0.0001****	0.08^S^
	Mean slope	Segment	1	70.87	< 0.0001****	0.71^L^
		Sleep dependent	2	144.53	< 0.0001****	2.44^L^
		Circadian	5	43.22	< 0.0001****	0.84^L^
		Segment ∗ Circadian	5	0.94	ns	
		Segment ∗ Sleep dependent	2	5.66	0.0045*	0.10^S^
		Sleep dependent ∗ Circadian	10	2.49	0.007	0.05^S^
	Maximum slope	Segment	1	82.06	< 0.0001****	0.79^L^
		Sleep dependent	2	259.39	< 0.0001****	4.22^L^
		Circadian	5	45.29	< 0.0001****	0.74^L^
		Segment ∗ Circadian	5	0.58	ns	
		Segment ∗ Sleep dependent	2	3.69	0.028	0.06^S^
		Sleep dependent ∗ Circadian	10	2.64	0.004*	0.05^S^
Positive	Duration	Segment	1	1.32	ns	
		Sleep dependent	2	23.46	< 0.0001****	0.38^L^
		Circadian	5	7.36	< 0.0001****	0.11^S^
		Segment ∗ Circadian	5	0.16	ns	
		Segment ∗ Sleep dependent	2	0.33	ns	
		Sleep dependent ∗ Circadian	10	4.03	< 0.0001****	0.07^S^
	Mean slope	Segment	1	9.43	0.003*	0.08^S^
		Sleep dependent	2	105.25	< 0.0001****	1.66^L^
		Circadian	5	18.57	< 0.0001****	0.28^M^
		Segment ∗ Circadian	5	0.22	ns	
		Segment ∗ Sleep dependent	2	0.35	ns	
		Sleep dependent ∗ Circadian	10	3.99	< 0.0001****	0.08^S^
	Maximum slope	Segment	1	188.79	< 0.0001****	1.74^L^
		Sleep dependent	2	200.75	< 0.0001****	3.22^L^
		Circadian	5	30.42	< 0.0001****	0.48^L^
		Segment ∗ Circadian	5	0.28	ns	
		Segment ∗ Sleep dependent	2	0.14	ns	
		Sleep dependent ∗ Circadian	10	3.68	< 0.0001****	0.07^S^

We assessed the effect of the ascending versus descending transitions between the positive and negative half-waves (initial versus final segment) for the circadian and sleep-dependent modulation of SW duration and slope measures. We found that both the circadian and sleep-dependent regulations were significantly present independent of the half-wave segment factor, which also yielded a significant effect on the studied SW parameters except for the duration of the positive half-waves. Most importantly, we confirmed that the circadian modulation of SW parameters was totally independent of the segment whereas the sleep-dependent modulation of the negative but not the positive SW half-wave was significantly modulated by the direction of the transition between the polarities. Results for both negative and positive half-waves are presented. The segment factor of the SW half-waves comprises the *initial* and the *final* segment. The sleep-dependent factor included thirds of the total sleep period (9 h 20 m). The circadian factor comprised 6 ∗ 60° bins. Degree of freedom (DF), *F* values, *P* values, effect size (*Cohen's f*^2^) of main effects, and interactions are indicated for each studied variables as returned from mixed model analyses of variances (*P < .005, ***P < .0005, ****P < .0001). Superscripts following effect size values indicate the magnitude of the effects [small(S): 0.02–0.15, medium (M): 0.15–0.35, large (L): > 0.35]. *P* values and effect sizes for non-significant effects are not indicated. Non-significant trends (< 0.05) are indicated.

Inline Supplementary Table S9

Inline Supplementary Fig. S4 and Table S6–S9 can be found online at http://dx.doi.org/10.1016/j.neuroimage.2015.05.012.

## Discussion

The data show for the first time that during NREM sleep, characteristics of slow waves (SWs) such as their slope, amplitude, incidence and duration are, to a considerable extent, modulated by the circadian phase at which sleep occurs and that the magnitude of the circadian variation relative to the sleep-dependent modulation, varies along the anterior–posterior brain axis. The data also confirm many previous finding such as the sleep dependent and circadian modulation of slow-wave activity (SWA) (Dijk and Czeisler, 1995), and the dominance of the sleep-dependent effect on SWA in frontal areas (Cajochen et al., 1999).

Although previous reports have provided some evidence for a time of day effect on SWs or SWA during human sleep (Baron and Reid, 2014; Dijk and Czeisler, 1995; Putilov et al., 2013; Zuccato et al., 2010), none of these studies demonstrated circadian variation in parameters of individually detected SWs collected within a gold standard protocol for the assessment of circadian variation. Furthermore, none of these studies assessed the topographical modulations of these circadian effects. Our analyses show that the circadian variation of slope parameters persists when controlling for the circadian variation in slow wave amplitude. Furthermore, the observed circadian variation is robust against the amplitude threshold or the frequency range used for SW detection, the EEG reference, the polarity or the transition direction between the polarities of the SW half-waves.

The circadian modulation of SW parameters is present independently of the circadian variations in total sleep time and NREM and REM sleep. Although in the forced desynchrony protocol, sleep is scheduled to occur at all circadian phases, thereby minimizing confounds of circadian and sleep-dependent effects, which are major when early and late sleep are compared, a small confound remains because NREM sleep duration is shorter at some circadian phases than at others (average minimum: 327 ± 29 min, average maximum: 393 ± 59 min). This can lead to differences in ‘sleep pressure’ at the end of each sleep episode that carries over to the next sleep period initiated 18 h and 40 min later. We have controlled for this confound by including in our statistical model the duration of NREM sleep and found that the circadian effect remained significant even when the circadian variation in sleep duration was taken into account (Inline Supplementary Table S2). We also deem it unlikely that the observed circadian variation in the SW characteristics during sleep are an ‘artefact’ of micro sleep or naps during the daytime, or large variations in activity during wakefulness. The participants were under continuous surveillance by a nurse or research officer during wakefulness. The data also show that the circadian modulation of SW parameters is particularly strong during the first third of the night, which is in line with a previous analysis which was limited to SWA (Dijk and Czeisler, 1995).

The circadian modulation of SW parameters is present independently of the circadian variations in total sleep time and NREM and REM sleep. Although in the forced desynchrony protocol, sleep is scheduled to occur at all circadian phases, thereby minimizing confounds of circadian and sleep-dependent effects, which are major when early and late sleep are compared, a small confound remains because NREM sleep duration is shorter at some circadian phases than at others (average minimum: 327 ± 29 min, average maximum: 393 ± 59 min). This can lead to differences in ‘sleep pressure’ at the end of each sleep episode that carries over to the next sleep period initiated 18 h and 40 min later. We have controlled for this confound by including in our statistical model the duration of NREM sleep and found that the circadian effect remained significant even when the circadian variation in sleep duration was taken into account (Inline Supplementary Table S2). We also deem it unlikely that the observed circadian variation in the SW characteristics during sleep are an ‘artefact’ of micro sleep or naps during the daytime, or large variations in activity during wakefulness. The participants were under continuous surveillance by a nurse or research officer during wakefulness. The data also show that the circadian modulation of SW parameters is particularly strong during the first third of the night, which is in line with a previous analysis which was limited to SWA (Dijk and Czeisler, 1995).

It may be argued that circadian variation in the intensity of wakefulness preceding sleep could have mediated the circadian variation in SWs during sleep. The intensity of wakefulness was standardized by carefully monitoring participants during wakefulness and by scheduling their activities which included five 40-min performance test batteries. We cannot exclude that variations in performance on the tests which are known to occur across the circadian cycle in forced desynchrony protocols (Dijk et al., 1992; Wyatt et al., 1999) could also contribute to circadian variation in SWs during subsequent sleep. This, however, is very unlikely to represent a major confound because in humans, compared to the effects of prior wakefulness, even the effect of variation in the intensity of wakefulness are rather small (De Bruin et al., 2002; Kattler et al., 1994).

Earlier studies established that SWs reflect alternating periods of synchronized neuronal firing and silence (Steriade et al., 1993b; Vyazovskiy et al., 2007, 2009). SWs were shown to be primarily local events with various cortical sources propagating across the cortex (Massimini et al., 2004; Murphy et al., 2009; Nir et al., 2011), which vary in parallel with sleep pressure, brain topography and sleep stage (Bersagliere and Achermann, 2010; Massimini et al., 2004; Menicucci et al., 2009; Riedner et al., 2007). SWs are hypothesized to play an important role in synaptic homeostasis (Tononi and Cirelli, 2006) and memory consolidation (Chauvette et al., 2012; Marshall et al., 2006; Ngo et al., 2013), modulate spindle and beta activity (Molle et al., 2002) and may be also linked to some trait-like individual differences related to chronotype (Mongrain et al., 2011), age (Carrier et al., 2011), cortical excitability and connectivity (Massimini et al., 2004). However, all these studies investigated SWs during baseline sleep or recovery sleep after sleep deprivation, which were scheduled according to the habitual nocturnal timing of the sleep period covering around only one third of the circadian clock. If we accept that the time course of SWs reflect changes in synaptic efficacy, and maintenance of synaptic homeostasis, and that these changes are essential to the recovery processes which occur during sleep, then the present findings imply that the neural plasticity related to the recovery function of sleep, as well as to sleep-dependent memory consolidation, is modulated by the time of day at which sleep occurs.

The circadian variation of SWs varied across the various parameters investigated. The duration and the slope parameters of SWs showed the strongest circadian modulation. This is unexpected because it has been suggested that slope parameters of SWs reflect firing synchronicity of neural groups is a key indicator of homeostatic sleep pressure and be more sensitive indicator than SWA (Esser et al., 2007; Riedner et al., 2007; Vyazovskiy et al., 2007, 2009), which is the traditional marker of homeostatic sleep pressure (Achermann and Borbely, 2003; Dijk, 2009).

On a neurophysiological level the duration of SWs has been linked to the time of transition from on to off states and vice versa of cortical neurons and we show here that this SW feature defining the slope of the SWs indicating of interneuronal synchronicity (Vyazovskiy et al., 2009) is under a considerable circadian modulation. Furthermore the relative independence of the slope from the amplitude of SWs is confirmed by our analyses showing that the intercept and the slope of the correlation between these two SW parameters are significantly different when comparing baseline night and the sleep episodes taken 12 h out of phase. In this context it is also relevant that when analysed in a forced desynchrony, spectral characteristics of the wake EEG show a circadian modulation. Importantly this showed that for the dominant EEG activity specific to wakefulness which indicates increased cortical arousal (8 to 20 Hz) the highest values are observed during the biological day and lowest values during the biological night (Cajochen et al., 2002; Golombek and Rosenstein, 2010). The data also show that the timing of the maximum across most studied SW measures is quite broad, rather peaks in the biological afternoon and does not coincide with the wake maintenance zone (Strogatz et al., 1987). The timing of the acrophase of the SW rhythm is similar to the timing of the maximum of the circadian rhythm in EEG alpha during scheduled wakefulness (Cajochen et al., 2002).

Overall, these data are consistent with the state-clock model of Frank and Cantera (2014) in which the circadian system and vigilance state (sleep vs. wakefulness) are considered to make synergistic contributions to synaptic plasticity. The increase in the incidence, amplitude, frequency and the slope during the circadian day may indicate a neurophysiological mode optimized for building up synaptic strength at that phase of the circadian cycle during which we are normally awake, whereas the opposite holds for the biological night when we normally are asleep, i.e. it represents a mode optimized for reducing overall synaptic strength.

It has often been described that during baseline sleep the largest decrease in SWA and SW incidence, amplitude and slope is observed in frontal derivations (Cajochen et al., 1999; Riedner et al., 2007). Our data demonstrate for the first time that this frontal predominance of the sleep-dependent decline is present at all circadian phases. Concomitantly, the circadian modulation of SW parameters showed very low variance (for SW incidence and amplitude) or no variance (for SW duration and slope) across derivations, indicating that the topographical effects on the effect sizes of the sleep-dependent component are not a simple consequence of topographical variation in EEG amplitude and associated changes in signal to noise ratio. The observation that the circadian modulation of certain SW parameters such as amplitude, duration and slope, outweighed the sleep-dependent modulation over certain brain areas such as the central and posterior regions, whereas the sleep-dependent modulation was dominant over the frontal region, may provide additional support for local sleep regulation (Huber et al., 2004; Krueger, 2011). It is possible that if the subjects had not performed the test-battery but instead had done nothing or something else during wakefulness, i.e. only do physical exercise, the topography of the circadian and sleep-dependent modulation would have been different. However, this is not invalidating the present demonstration of a circadian modulation of SWs.

Within both the negative and positive half-waves, we can distinguish between slope of the ascending and descending segments, reflecting transition from neural up-state to down-state, and vice-versa (Esser et al., 2007). Importantly, the sleep-dependent variation of slope measures was modulated by the polarity and the segment of the transition between the SW half-waves. However, the circadian variation was robust across these factors suggesting that the observed circadian modulation is not dependent on parameters defining the SWs. This is further supported by the observation that the sleep-dependent and circadian regulation of SW parameters was present regardless of the amplitude threshold and frequency range used for SW detection or the EEG reference used for the analyses. Although these factors influenced the absolute values of the studied SW parameters, and their sleep-dependent modulation, the circadian regulation of the slope parameters was unaffected suggesting that this is a robust physiological mechanism affecting slow oscillatory activity of the human EEG.

From our analyses we cannot deduce at which level the observed circadian modulation emerges. It may represent a circadian modulation of neuromodulators driven by the SCN (Ross et al., 2014) , maybe those related to the circadian variation in REM sleep, or a direct circadian modulation of synaptic efficacy/local connectivity, perhaps driven by local rhythms in clock genes and clock mechanisms, which are abundant throughout the central nervous system (Li et al., 2013). There is emerging evidence that the circadian system modulates aspects of extra-SCN neurons independent of vigilance states, and even independent of SCN input (Guilding and Piggins, 2007). This sleep-independent circadian modulation extends to brain functions such as synapse formation and maintenance (Liston et al., 2013), and many aspects of performance and cognition (Wang et al., 2009). Our data were collected in carefully selected healthy young people and to what extent these observations can be generalized to other age groups or patient populations, remains to be established.

Although our data are not at variance with the notion that SWs are to a considerable extent under control of the duration of prior wakefulness and sleep, they do establish that there is a sleep-duration-independent circadian contribution that varies with EEG derivation. Previously it has been established that the duration of NREM sleep, characteristics of NREM EEG power spectra and sleep spindles (Dijk and Czeisler, 1995; Dijk et al., 1997; Wei et al., 1999) are all under direct circadian control. Together with the current data, this implies indeed that all main EEG characteristic of NREM sleep are under circadian control.

We do not know to what extent this circadian modulation varies between individuals or with age or cognitive status. Our studied group consisted of young healthy participants. There are, however, no reasons to assume that these effects are specific to this group only. Elucidating the underlying neurophysiological and molecular mechanisms of this circadian modulation holds promise for a more complete understanding of sleep function, the temporal organization of vigilance states, which in our species is very much dependent on circadian phase, and the dependency of the execution of sleep's recovery function on the time of day at which sleep is taken.

## Conclusions

Slow waves represent the hallmark of human sleep involved in the recovery function of sleep related to changes in cellular metabolism and synaptic homeostasis. However, our extensive dataset challenge the widespread notion that slow-oscillatory activity in sleep is exclusively regulated in a sleep-dependent manner and is not modulated by circadian processes. We show that circadian phase at which sleep occurs has a major effect on slow waves independent of sleep history. Furthermore, the circadian modulation of slow waves outweighs the sleep dependent effect in multiple brain regions. Thus, the circadian regulation of sleep extends beyond the modulation of sleep efficiency, REM sleep and sleep spindle activity, and also acts on the core neural functions of the sleep-dependent recovery as reflected in slow oscillatory activity. Overall, the data imply that circadian phase at which sleep occurs may be far more critical for our neural and cognitive integrity than previously assumed. This warrants further studies of the interaction of circadian phase and vigilance state.

## Funding

This research was supported by a Biotechnology and Biological Sciences Research Council grant (BB/F022883 to DJ.D. and others). DJD is supported by a Royal Society-Wolfson Research Merit Award.

## Figures and Tables

**Table 1:** Summary of main effects and interactions of brain topography and time in sleep period on the studied SW parameters during baseline night.

SW parameters	Segment	Brain topography (T)				Sleep dependent effect (H)				Interaction			
		*DF*	*F* value	*P* value	Cohen's *f*^2^	*DF*	*F* value	*P* value	*Cohen*'*s f*^2^	*DF*	*F* value	*P* value	*Cohen*'*s f*^2^
SWA^†^(μV^2^ ∗ s)		2	48.1	< 0.0001****	1.44^L^	3	61.3	< 0.0001****	1.50^L^	6	13.4	< 0.0001****	0.57^L^
Incidence (SW/min)^†^		2	305.5	< 0.0001****	12.09^L^	3	295.9	< 0.0001****	8.58^L^	6	109.0	< 0.0001****	4.71^L^
Amplitude (μV)^†^		2	152.8	< 0.0001****	5.87^L^	3	91.2	< 0.0001****	2.54^L^	6	55.4	< 0.0001****	2.36^L^
Duration (s)	Both	2	340.2	< 0.0001****	13.27^L^	3	34.1	< 0.0001****	0.98^L^	6	9.2	< 0.0001****	0.39^L^
	Initial^†^	2	108.6	< 0.0001****	4.15^L^	3	29.9	< 0.0001****	0.81^L^	6	8.7	< 0.0001****	0.37^L^
	Final^†^	2	448.8	< 0.0001****	17.48^L^	3	42.0	< 0.0001****	1.23^L^	6	9.9	< 0.0001****	0.43^L^
Mean slope (μV/s)	Both	2	334.4	< 0.0001****	12.87^L^	3	78.9	< 0.0001****	2.15^L^	6	52.8	< 0.0001****	2.25^L^
	Initial^†^	2	231.0	< 0.0001****	8.92^L^	3	61.8	< 0.0001****	1.74^L^	6	48.0	< 0.0001****	2.05^L^
	Final^†^	2	402.5	< 0.0001****	15.40^L^	3	84.1	< 0.0001****	2.30^L^	6	47.3	< 0.0001****	2.01^L^
Maximum slope (μV/s)	Both	2	250.3	< 0.0001****	9.68^L^	3	92.6	< 0.0001****	2.53^L^	6	58.8	< 0.0001****	2.51^L^
	Initial	2	158.5	< 0.0001****	6.13^L^	3	74.0	< 0.0001****	2.05^L^	6	54.0	< 0.0001****	2.31^L^
	Final	2	332.7	< 0.0001****	12.80^L^	3	101.3	< 0.0001****	2.78^L^	6	54.5	< 0.0001****	2.33^L^

Results for slow wave (SW) negative half-waves are presented. The brain topography factor comprises three main brain regions each including weighted averages over the Frontal (Fp1, Fp2, C3, C4), Central (C3, C4, T3, T4), and Posterior (P3, P4, O1, O2) areas. The time in sleep period factor comprises 2-hourly intervals of the first 8 h of the sleep period. The Segment variable indicates the descending (initial) or the ascending (final) phase of the slow wave negative half waves. Absolute mean values, standard deviations (SD), degree of freedom (DF), *F* values, *P* values, effect size (*Cohen*'*s f*^2^) of main effects, and interactions are indicated for all studied variables (****P < .0001). Superscripts following variable names (†) indicate SW parameters graphically represented in Fig. 2. Superscripts following effect size values indicate the magnitude of the effects size [small (S): 0.02–0.15, medium (M): 0.15–0.35, large (L): > 0.35]. *P* values and effect sizes for non-significant effects are not indicated. Non-significant trends (P < 0.05) are indicated.

**Table 2:** Summary of sleep dependent and circadian main effects and interactions.

SW parameters	Segment	Mean	SD	Sleep dependent effect (H)				Circadian effect (C)				Interaction (H ∗ C)			
				*DF*	*F value*	*P* value	*Cohen*'*s f*^2^	*DF*	*F value*	*P* value	*Cohen*'*s f*^2^	*DF*	*F value*	*P* value	*Cohen's f*^2^
SWA (μV^2^*s)		163.35	53.83	2	348.2	< .0001****	1.98^L^	5	5.36	< .0001****	0.06^S^	10	0.48	ns	
Incidence (SW/min)^†^		7.50	2.10	2	557.9	< .0001****	2.64^L^	5	9.77	< .0001****	0.09^S^	10	1.68	ns	
Amplitude (μV) ^†^		56.00	1.80	2	292.7	< .0001****	1.73^L^	5	9.33	< .0001****	0.10^S^	10	2.34	0.01*	0.04^S^
Duration (sec)^†^ Mean slope (μV/s)†	Both	0.47	0.02	2	17.2	< .0001****	0.09^S^	5	6.47	< .0001****	0.06^S^	10	2.05	0.027*	0.03^S^
	Initial	0.24	0.01	2	30.6	< .0001****	0.15^S^	5	3.58	.0034*	0.03^S^	10	2.75	0.003**	0.04^S^
	Final	0.23	0.01	2	4.7	.01	0.03 ^S^	5	6.62	< .0001****	0.06^S^	10	1.07	ns	
	Both	290.70	19.20	2	99.7	< .0001****	0.57^L^	5	17.89	< .0001****	0.17^M^	10	1.30	ns	
	Initial	283.80	18.70	2	66.1	< .0001****	0.35^M^	5	10.78	< .0001****	0.10^S^	10	2.08	0.024*	0.03^S^
	Final	297.60	20.00	2	112.2	< .0001****	0.74^L^	5	17.48	< .0001****	0.18^M^	10	0.65	ns	
Maximum slope ^†^ (μV/s)	Both	489.60	33.10	2	182.8	< .0001****	1.07^L^	5	19.43	< .0001****	0.17^M^	10	1.46	ns	
	Initial	496.70	34.90	2	120.7	< .0001****	0.67^L^	5	11.18	< .0001****	0.09^S^	10	1.90	0.042*	0.03^S^
	Final	482.50	31.70	2	215.7	< .0001****	1.41^L^	5	21.60	< .0001****	0.21^M^	10	1.06	ns	

Results for negative half waves are presented as averaged across all EEG derivations. The sleep-dependent factor comprises thirds of the total sleep period (9 h 20 m). The circadian factor comprises 6 ∗ 60° (~ 4-hourly) bins. The Segment variable indicates the descending (initial) or the ascending (final) phase of the slow wave (SW) negative half waves. When the Segment column indicates ‘Both’ presented values are obtained from the summation (Duration) or the averaging (slope measures) of the values corresponding to the initial and final SW half-wave segments. Absolute mean values, standard deviations (SD), degree of freedom (DF), *F* values, *P* values, effect size (*Cohen's f^2^*) of main effects, and interactions are indicated for each studied variables as returned from mixed model analyses of variances (*P < .005, **P < .001, ****P < .0001). Superscripts following variable (†) names indicate SW parameters graphically represented in Fig. 4. Superscripts following effect size values indicate the magnitude of the effects size [small(S): 0.02–0.15, medium (M): 0.15–0.35, large (L): > 0.35]. *P* values and effect sizes for non-significant effects are not indicated. Non-significant trends (*P* < 0.05) are indicated.
