# Supplementary material for: Circadian regulation of slow waves in human sleep: Topographical aspects
Source: Neuroimage. 2015 Aug 1;116:123–34. doi: 10.1016/j.neuroimage.2015.05.012 (PMC4503801; doi:10.1016/j.neuroimage.2015.05.012)
Supplement: Inline Supplementary Table S1 [file mmc1.doc]

**Table S1.** Representative example of correlations between individual slow measures measured at baseline night (N= 3531 Slow waves, BB0214, Female, 31 years, EEG channel: C3)

| **Variable** | **Amplitude** | **Duration initial segment** | **Duration final segment** | **Mean slope initial segment** | **Mean slope final segment** | **Maximum slope initial segment** | **Maximum slope final segment** |
| --- | --- | --- | --- | --- | --- | --- | --- |
| **Amplitude** | 1 | 0.16475 | 0.08064 | 0.31887 | 0.3662 | 0.41186 | 0.41591 |
|  | _ | **** | **** | **** | **** | **** | **** |
| **Duration initial segment** | 0.16475 | 1 | -0.23783 | -0.86369 | 0.28204 | -0.42147 | 0.18372 |
|  | **** | _ | **** | **** | **** | **** | **** |
| **Duration final segment** | 0.08064 | -0.23783 | 1 | 0.25755 | -0.87838 | 0.15104 | -0.48273 |
|  | **** | **** | _ | **** | **** | **** | **** |
| **Mean slope initial segment** | 0.31887 | -0.86369 | 0.25755 | 1 | -0.0698 | 0.64015 | 0.0451 |
|  | **** | **** | **** | _ | *** | **** | ns |
| **Mean slope final segment** | 0.3662 | 0.28204 | -0.87838 | -0.0698 | 1 | 0.06831 | 0.68307 |
|  | **** | **** | **** | **** | _ | *** | **** |
| **Maximum slope initial segment** | 0.41186 | -0.42147 | 0.15104 | 0.64015 | 0.06831 | 1 | 0.16798 |
|  | **** | **** | **** | **** | **** | _ | **** |
| **Maximum slope final segment** | 0.41591 | 0.18372 | -0.48273 | 0.0451 | 0.68307 | 0.16798 | 1 |
|  | **** | **** | **** | ns | **** | **** | _ |

Spearman Rho and significance value are indicated for the studied parameters of all negative half waves of all detected SWs (N= 3531) as measured during the baseline night. (*** *P* < .0005, **** *P* <.0001).
