# Supplementary material for: Circadian regulation of slow waves in human sleep: Topographical aspects
Source: Neuroimage. 2015 Aug 1;116:123–34. doi: 10.1016/j.neuroimage.2015.05.012 (PMC4503801; doi:10.1016/j.neuroimage.2015.05.012)
Supplement: Inline Supplementary Table S2 [file mmc2.doc]

**Table S2.** Summary of main effects and interactions of factors thirds of the night and sleep episodes scheduled around the circadian clock as well as main effects of covariates REM and NREM duration during the preceding sleep episodes on the studied SW parameters as measured during the forced desynchrony

| SW parameter | Segment | 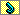Effect | *DF* | *F* value | *P* value |  | Cohen’s *f^2^* |  |
| --- | --- | --- | --- | --- | --- | --- | --- | --- |
| Incidence |  | Thirds of the night | 2 | 604.54 | <.0001 | **** | 23.34 | L |
|  |  | Sleep episode | 6 | 20.01 | <.0001 | **** | 0.66 | L |
|  |  | Sleep episode *Thirds of the night | 12 | 4.86 | <.0001 | **** | 0.20 | M |
|  |  | REM duration | 1 | 0.57 | ns |  |  |  |
|  |  | NREM duration | 1 | 0.86 | ns |  |  |  |
| Amplitude |  | Thirds of the night | 2 | 174.61 | <.0001 | **** | 6.79 | L |
|  |  | Sleep episode | 6 | 14.34 | <.0001 | **** | 0.47 | L |
|  |  | Sleep episode *Thirds of the night | 12 | 3.79 | <.0001 | **** | 0.16 | M |
|  |  | REM duration | 1 | 2.76 | ns |  |  |  |
|  |  | NREM duration | 1 | 1 | ns |  |  |  |
| Duration | Both | Thirds of the night | 2 | 21.57 | <.0001 | **** | 0.81 | L |
|  |  | Sleep episode | 6 | 10.85 | <.0001 | **** | 0.34 | M |
|  |  | Sleep episode *Thirds of the night | 12 | 3.63 | <.0001 | **** | 0.15 | M |
|  |  | REM duration | 1 | 7.77 | 0.006 |  | 0.04 | S |
|  |  | NREM duration | 1 | 0.35 | ns |  |  |  |
|  | Initial | Thirds of the night | 2 | 47.75 | <.0001 | **** | 1.77 | L |
|  |  | Sleep episode | 6 | 11.33 | <.0001 | **** | 0.36 | L |
|  |  | Sleep episode *Thirds of the night | 12 | 4.89 | <.0001 | **** | 0.20 | M |
|  |  | REM duration | 1 | 7.54 | 0.007 |  | 0.04 | S |
|  |  | NREM duration | 1 | 0.38 | ns |  |  |  |
|  | Final | Thirds of the night | 2 | 7.61 | 0.001 | * | 0.29 | L |
|  |  | Sleep episode | 6 | 8.52 | <.0001 | **** | 0.28 | M |
|  |  | Sleep episode *Thirds of the night | 12 | 2.36 | 0.007 |  | 0.1 | S |
|  |  | REM duration | 1 | 6.95 | 0.009 |  | 0.04 | S |
|  |  | NREM duration | 1 | 0.61 | ns |  |  |  |
| Mean Slope | Both | Thirds of the night | 2 | 89.72 | <.0001 | **** | 3.51 | L |
|  |  | Sleep episode | 6 | 21.82 | <.0001 | **** | 0.68 | L |
|  |  | Sleep episode *Thirds of the night | 12 | 7.14 | <.0001 | **** | 0.30 | M |
|  |  | REM duration | 1 | 6.11 | 0.014 |  | 0.03 | S |
|  |  | NREM duration | 1 | 0.87 | ns |  |  |  |
|  | Initial | Thirds of the night | 2 | 345.05 | <.0001 | **** | 3.19 | L |
|  |  | Sleep episode | 6 | 20.94 | <.0001 | **** | 0.99 | L |
|  |  | Sleep episode *Thirds of the night | 12 | 4.6 | <.0001 | **** | 0.17 | M |
|  |  | REM duration | 1 | 4.01 | 0.047 |  | 0.02 | S |
|  |  | NREM duration | 1 | 0.38 | ns |  |  |  |
|  | Final | Thirds of the night | 2 | 57.31 | <.0001 | **** | 2.25 | L |
|  |  | Sleep episode | 6 | 17.92 | <.0001 | **** | 0.58 | L |
|  |  | Sleep episode *Thirds of the night | 12 | 4.84 | <.0001 | **** | 0.21 | M |
|  |  | REM duration | 1 | 6.52 | 0.012 |  | 0.03 | L |
|  |  | NREM duration | 1 | 1.54 | ns |  |  |  |
| Maximum slope | Both | Thirds of the night | 2 | 120.97 | <.0001 | **** | 4.78 | L |
|  |  | Sleep episode | 6 | 22.76 | <.0001 | **** | 0.71 | L |
|  |  | Sleep episode *Thirds of the night | 12 | 8.64 | <.0001 | **** | 0.37 | L |
|  |  | REM duration | 1 | 4.2 | 0.042 |  | 0.02 | L |
|  |  | NREM duration | 1 | 0.66 | ns |  |  |  |
|  | Initial | Thirds of the night | 2 | 152.42 | <.0001 | **** | 5.98 | L |
|  |  | Sleep episode | 6 | 22.66 | <.0001 | **** | 0.71 | L |
|  |  | Sleep episode *Thirds of the night | 12 | 9.69 | <.0001 | **** | 0.41 | L |
|  |  | REM duration | 1 | 2.93 | ns |  |  |  |
|  |  | NREM duration | 1 | 0.11 | ns |  |  |  |
|  | Final | Thirds of the night | 2 | 86.29 | <.0001 | **** | 3.43 | L |
|  |  | Sleep episode | 6 | 19.72 | <.0001 | **** | 0.64 | L |
|  |  | Sleep episode *Thirds of the night | 12 | 6.03 | <.0001 | **** | 0.26 | M |
|  |  | REM duration | 1 | 4.27 | 0.04 |  | 0.02 | S |
|  |  | NREM duration | 1 | 1.18 | ns |  |  |  |

Results for negative half-waves are presented. The Thirds of the night factor includes thirds of the total sleep period (9h20m). The sleep episode factor comprises of 7 sleep episodes scheduled around the circadian clock. REM duration and NREM duration are continuous covariates included in the model and indicate the sleep duration measured always during the previous sleep episode as compared the SW parameters. Segment variable indicates the descending (initial) or the ascending (final) phase of the slow wave (SW) negative half waves. Degree of freedom (DF), *F* values, *P* values, effect size (*Cohen’s f ^2^*) of main effects, and interactions are indicated for each studied variables as returned from mixed model analyses of variances ( * *P* < .005, ** *P* < .001, *** *P* < .0005, **** *P* <.0001). Superscripts following effect size values indicate the magnitude of the effects size [small(S): 0.02-0.15, medium (M): 0.15-0.35, large (L): >0.35]. *P* values and effect sizes for non-significant effects are not indicated. Non-significant trends (<0.05) are indicated.
