# Supplementary material for: Circadian regulation of slow waves in human sleep: Topographical aspects
Source: Neuroimage. 2015 Aug 1;116:123–34. doi: 10.1016/j.neuroimage.2015.05.012 (PMC4503801; doi:10.1016/j.neuroimage.2015.05.012)
Supplement: Inline Supplementary Table S3 [file mmc3.doc]

**Table S3.** Summary of main effects and interactions of the brain topography, sleep dependent and circadian factors on the studied SW parameters as measured during the forced desynchrony

| SW parameter | Segment | 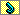Effect | *DF* | *F* value | *P* value |  | Cohen’s *f^2^* |  |
| --- | --- | --- | --- | --- | --- | --- | --- | --- |
| Incidence |  | Topography | 2 | 697.44 | <0.0001 | **** | 6.98 | L |
|  |  | Sleep dependent | 2 | 728.76 | <0.0001 | **** | 7.54 | L |
|  |  | Circadian | 5 | 35.44 | <0.0001 | **** | 0.36 | L |
|  |  | Topography*Sleep dependent | 4 | 47.46 | <0.0001 | **** | 0.93 | L |
|  |  | Topography*Circadian | 10 | 3.7 | <0.0001 | **** | 0.07 | S |
|  |  | Sleep dependent*Circadian | 10 | 6.81 | <0.0001 | **** | 0.08 | S |
| Amplitude |  | Topography | 2 | 519.88 | <0.0001 | **** | 5.07 | L |
|  |  | Sleep dependent | 2 | 195.52 | <0.0001 | **** | 2.12 | L |
|  |  | Circadian | 5 | 10.22 | <0.0001 | **** | 0.11 | S |
|  |  | Topography*Sleep dependent | 4 | 33.24 | <0.0001 | **** | 0.68 | L |
|  |  | Topography*Circadian | 10 | 2.01 | 0.0303 |  | 0.04 | S |
|  |  | Sleep dependent*Circadian | 10 | 3.43 | 0.0002 | *** | 0.04 | S |
| Duration | Initial | Topography | 2 | 139.29 | <0.0001 | **** | 1.35 | L |
|  |  | Sleep dependent | 2 | 33.82 | <0.0001 | **** | 0.36 | L |
|  |  | Circadian | 5 | 11.61 | <0.0001 | **** | 0.12 | S |
|  |  | Topography*Sleep dependent | 4 | 4.66 | 0.0013 | * | 0.09 | S |
|  |  | Topography*Circadian | 10 | 0.25 | ns |  |  |  |
|  |  | Sleep dependent*Circadian | 10 | 6.46 | <0.0001 | **** | 0.08 | S |
|  | Final | Topography | 2 | 845.16 | <0.0001 | **** | 8.36 | L |
|  |  | Sleep dependent | 2 | 52.3 | <0.0001 | **** | 0.56 | L |
|  |  | Circadian | 5 | 20.77 | <0.0001 | **** | 0.25 | M |
|  |  | Topography*Sleep dependent | 4 | 7.86 | <0.0001 | **** | 0.16 | M |
|  |  | Topography*Circadian | 10 | 0.35 | ns |  |  |  |
|  |  | Sleep dependent*Circadian | 10 | 3.58 | 0.0001 | *** | 0.05 | S |
| Mean Slope | Initial | Topography | 2 | 815.92 | <0.0001 | **** | 7.90 | L |
|  |  | Sleep dependent | 2 | 76.54 | <0.0001 | **** | 0.80 | L |
|  |  | Circadian | 5 | 20.22 | <0.0001 | **** | 0.21 | M |
|  |  | Topography*Sleep dependent | 4 | 26.42 | <0.0001 | **** | 0.52 | L |
|  |  | Topography*Circadian | 10 | 0.97 | ns |  |  |  |
|  |  | Sleep dependent*Circadian | 10 | 4.06 | <0.0001 | **** | 0.05 | S |
|  | Final | Topography | 2 | 1371 | <0.0001 | **** | 14.37 | L |
|  |  | Sleep dependent | 2 | 153.57 | <0.0001 | **** | 1.63 | L |
|  |  | Circadian | 5 | 29.9 | <0.0001 | **** | 0.31 | M |
|  |  | Topography*Sleep dependent | 4 | 10.66 | <0.0001 | **** | 0.21 | M |
|  |  | Topography*Circadian | 10 | 1.68 | ns |  |  |  |
|  |  | Sleep dependent*Circadian | 10 | 2.2 | 0.0162 |  | 0.03 | S |
| Maximum slope | Initial | Topography | 2 | 611.73 | <0.0001 | **** | 5.88 | L |
|  |  | Sleep dependent | 2 | 107.72 | <0.0001 | **** | 1.13 | L |
|  |  | Circadian | 5 | 14.03 | <0.0001 | **** | 0.14 | S |
|  |  | Topography*Sleep dependent | 4 | 29.01 | <0.0001 | **** | 0.58 | L |
|  |  | Topography*Circadian | 10 | 1.4 | ns |  |  |  |
|  |  | Sleep dependent*Circadian | 10 | 3.45 | 0.0002 | *** | 0.04 | S |
|  | Final | Topography | 2 | 1410 | <0.0001 | **** | 14.67 | L |
|  |  | Sleep dependent | 2 | 244.1 | <0.0001 | **** | 2.55 | L |
|  |  | Circadian | 5 | 25.74 | <0.0001 | **** | 0.26 | M |
|  |  | Topography*Sleep dependent | 4 | 13.2 | <0.0001 | **** | 0.26 | M |
|  |  | Topography*Circadian | 10 | 1.77 | ns |  |  |  |
|  |  | Sleep dependent*Circadian | 10 | 2.6 | 0.0042 | * | 0.03 | S |

Results for negative half-waves are presented. The brain topography factor comprises three main brain regions each including weighted averages over the Frontal (Fp1, Fp2, C3, C4), Central (C3, C4, T3, T4), and Posterior (P3, P4, O1, O2) areas. The sleep-dependent factor includes thirds of the total sleep period (9h20m). The circadian factor comprises of 6*60 degree (~ 4-hourly) bins. The Segment variable indicates the descending (initial) or the ascending (final) phase of the slow wave (SW) negative half waves. Degree of freedom (DF), *F* values, *P* values, effect size (*Cohen’s f ^2^*) of main effects, and interactions are indicated for each studied variables as returned from mixed model analyses of variances ( * *P* < .005, ** *P* < .001, *** *P* < .0005, **** *P* <.0001). Superscripts following effect size values indicate the magnitude of the effects size [small(S): 0.02-0.15, medium (M): 0.15-0.35, large (L): >0.35]. *P* values and effect sizes for non-significant effects are not indicated. Non-significant trends (<0.05) are indicated.
