# Supplementary material for: Circadian regulation of slow waves in human sleep: Topographical aspects
Source: Neuroimage. 2015 Aug 1;116:123–34. doi: 10.1016/j.neuroimage.2015.05.012 (PMC4503801; doi:10.1016/j.neuroimage.2015.05.012)
Supplement: Inline Supplementary Table S4 [file mmc4.doc]

**Table S4. Summary of main effects of the sleep dependent and circadian factors on the studied SO parameters within each main brain region as measured during the forced desynchrony**

| 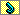SW parameters | Segment | Regions | Mean | SD |  | Sleep dependent effect (H) | | | |  |  | Circadian effect (C) | | | |  |
| --- | --- | --- | --- | --- | --- | --- | --- | --- | --- | --- | --- | --- | --- | --- | --- | --- |
|  |  |  |  |  | *DF* | *F* value | *P* value |  | Cohen’s  *f^2^* |  | *DF* | *F* Value | *P* value |  | Cohen’s *f^2^* |  |
| Incidence |  | Frontal^CP^ | 11.62 | 2.58 | 2 | 511.3 | <0.0001 | **** | 2.46 | L | 5 | 9.5 | <0.0001 | **** | 0.09 | S |
| (SO/min) |  | Central^P^ | 7.28 | 2.28 | 2 | 487.7 | <0.0001 | **** | 2.39 | L | 5 | 8.4 | <0.0001 | **** | 0.08 | S |
|  |  | Posterior | 3.56 | 1.88 | 2 | 170.4 | <0.0001 | **** | 1.22 | L | 5 | 3.1 | 0.0087 |  | 0.03 | S |
| Amplitude |  | Frontal^P^ | 57.95 | 2.13 | 2 | 300.6 | <0.0001 | **** | 1.69 | L | 5 | 8.2 | <0.0001 | **** | 0.08 | S |
| (µV) |  | Central | 55.03 | 2.32 | 2 | 170.1 | <0.0001 | **** | 0.98 | L | 5 | 5.6 | <0.0001 | **** | 0.06 | S |
|  |  | Posterior | 50.92 | 2.34 | 2 | 6.6 | 0.0015 | * | 0.04 | S | 5 | 0.9 | ns |  |  |  |
| Duration | Both | Frontal^CP^ | 0.45 | 0.02 | 2 | 20.6 | <0.0001 | **** | 0.10 | S | 5 | 6.1 | <0.0001 | **** | 0.06 | S |
| (sec) |  | Central^P^ | 0.50 | 0.02 | 2 | 13.5 | <0.0001 | **** | 0.07 | S | 5 | 6.5 | <0.0001 | **** | 0.06 | S |
|  |  | Posterior | 0.52 | 0.03 | 2 | 5.3 | 0.0053 |  | 0.03 | S | 5 | 6.1 | <0.0001 | **** | 0.05 | S |
|  | Initial | Frontal^CP^ | 0.23 | 0.01 | 2 | 27.0 | <0.0001 | **** | 0.14 | S | 5 | 2.4 | 0.036 |  | 0.02 | S |
|  |  | Central | 0.25 | 0.01 | 2 | 21.5 | <0.0001 | **** | 0.11 | S | 5 | 3.2 | 0.0069 |  | 0.03 | S |
|  |  | Posterior | 0.25 | 0.01 | 2 | 24.6 | <0.0001 | **** | 0.14 | S | 5 | 4.9 | 0.0002 | **** | 0.05 | S |
|  | Final | Frontal^CP^ | 0.22 | 0.01 | 2 | 13.9 | <0.0001 | **** | 0.08 | S | 5 | 8.0 | <0.0001 | **** | 0.08 | S |
|  |  | Central^P^ | 0.25 | 0.01 | 2 | 4.6 | 0.0108 |  | 0.03 | S | 5 | 6.1 | <0.0001 | **** | 0.06 | S |
|  |  | Posterior | 0.27 | 0.01 | 2 | 33.1 | <0.0001 | **** | 0.22 | M | 5 | 4.1 | 0.0012 | * | 0.04 | S |
| Mean slope | Both | Frontal^CP^ | 318.3 | 23.72 | 2 | 170.9 | <0.0001 | **** | 1.02 | L | 5 | 17.1 | <0.0001 | **** | 0.16 | M |
| (µV)/sec |  | Central^P^ | 271.0 | 22.53 | 2 | 58.9 | <0.0001 | **** | 0.33 | M | 5 | 12.4 | <0.0001 | **** | 0.11 | S |
|  |  | Posterior | 235.6 | 24.14 | 2 | 9.7 | <0.0001 | **** | 0.07 | S | 5 | 2.8 | 0.0165 |  | 0.02 | S |
|  | Initial | Frontal^CP^ | 306.6 | 22.41 | 2 | 134.7 | <0.0001 | **** | 0.75 | L | 5 | 10.9 | <0.0001 | **** | 0.10 | S |
|  |  | Central^P^ | 267.0 | 21.80 | 2 | 28.0 | <0.0001 | **** | 0.15 | S | 5 | 6.3 | <0.0001 | **** | 0.06 | S |
|  |  | Posterior | 239.2 | 23.81 | 2 | 6.9 | 0.0011 | * | 0.04 | S | 5 | 3.1 | 0.0097 |  | 0.03 | S |
|  | Final | Frontal^CP^ | 330.0 | 25.58 | 2 | 172.4 | <0.0001 | **** | 1.09 | L | 5 | 17.4 | <0.0001 | **** | 0.16 | S |
|  |  | Central^P^ | 275.0 | 23.58 | 2 | 75.1 | <0.0001 | **** | 0.48 | L | 5 | 13.2 | <0.0001 | **** | 0.14 | S |
|  |  | Posterior | 232.0 | 25.28 | 2 | 43.8 | <0.0001 | **** | 0.35 | M | 5 | 2.6 | 0.0235 |  | 0.02 | S |
| Max slope | Both | Frontal^CP^ | 527.4 | 39.50 | 2 | 260.7 | <0.0001 | **** | 1.54 | L | 5 | 18.6 | <0.0001 | **** | 0.16 | M |
| (µV)/sec |  | Central^P^ | 464.3 | 38.47 | 2 | 106.7 | <0.0001 | **** | 0.63 | L | 5 | 12.0 | <0.0001 | **** | 0.10 | S |
|  |  | Posterior | 409.5 | 42.49 | 2 | 14.5 | <0.0001 | **** | 0.11 | S | 5 | 1.3 | ns |  |  |  |
|  | Initial | Frontal^CP^ | 530.4 | 40.66 | 2 | 223.0 | <0.0001 | **** | 1.25 | L | 5 | 13.3 | <0.0001 | **** | 0.12 | S |
|  |  | Central^P^ | 473.3 | 39.94 | 2 | 53.9 | <0.0001 | **** | 0.31 | M | 5 | 6.8 | <0.0001 | **** | 0.06 | S |
|  |  | Posterior | 427.1 | 45.02 | 2 | 3.2 | 0.0423 |  | 0.02 | S | 5 | 1.5 | ns |  |  |  |
|  | Final | Frontal^CP^ | 524.4 | 38.91 | 2 | 254.4 | <0.0001 | **** | 1.61 | L | 5 | 18.6 | <0.0001 | **** | 0.17 | M |
|  |  | Central^P^ | 455.3 | 37.51 | 2 | 150.3 | <0.0001 | **** | 0.91 | L | 5 | 15.7 | <0.0001 | **** | 0.16 | M |
|  |  | Posterior | 392.0 | 41.63 | 2 | 61.8 | <0.0001 | **** | 0.53 | L | 5 | 1.7 | ns |  |  |  |

Results for negative half-waves are presented. The main brain regions each included weighted averages over the Frontal (Fp1, Fp2, C3, C4), Central (C3, C4, T3, T4), and Posterior (P3, P4, O1, O2) areas. The sleep-dependent factor comprises thirds of the total sleep period (9 h 20 m). The circadian factor comprised 6*60 degree (~ 4-hourly) bins. The Segment variable indicates the descending (initial) or the ascending (final) phase of the slow wave (SW) negative half waves. When the Segment column indicates ‘Both’ presented values are obtained from the summation (Duration) or the averaging (slope measures) of the values corresponding to the initial and final SW half-wave segments. Absolute mean values, standard deviations, degree of freedom (DF), *F* values, *P* values, effect size (*Cohen’s f ^2^*) of main effects, and interactions are indicated for each studied variables as returned from mixed model analyses of variances ( * *P* < .005, ** *P* < .001, *** *P* < .0005, **** *P* <.0001). Superscripts following effect size values indicate the magnitude of the effects size [small(S): 0.02-0.15, medium (M): 0.15-0.35, large (L): >0.35]. *P* values and effect sizes for non-significant effects are not indicated. Non-significant trends (<0.05) are indicated.
