# Supplementary material for: Circadian regulation of slow waves in human sleep: Topographical aspects
Source: Neuroimage. 2015 Aug 1;116:123–34. doi: 10.1016/j.neuroimage.2015.05.012 (PMC4503801; doi:10.1016/j.neuroimage.2015.05.012)
Supplement: Inline Supplementary Table S5 [file mmc5.doc]

**Table S5.** Summary of main effects and interactions of SW detection amplitude threshold, sleep dependent and circadian factors on the studied SW parameters as measured during the forced desynchrony

| SW parameter | Segment | Effect | DF | F value | P value |  | Cohen's *f ^2^* |  |
| --- | --- | --- | --- | --- | --- | --- | --- | --- |
| Incidence |  | Amplitude threshold | 1 | 27334.6 | <0.0001 | **** | 206.87 | L |
|  |  | Sleep dependent | 2 | 49.88 | <0.0001 | **** | 0.87 | L |
|  |  | Circadian | 5 | 11.28 | <0.0001 | **** | 0.18 | M |
|  |  | Amplitude threshold*Circadian | 5 | 4.07 | 0.0013 | * | 0.06 | S |
|  |  | Amplitude threshold*Sleep dependent | 2 | 81.61 | <0.0001 | **** | 1.42 | L |
|  |  | Sleep dependent*Circadian | 10 | 1.77 | ns |  |  |  |
| Amplitude |  | Amplitude threshold | 1 | 42229.4 | <0.0001 | **** | 336.91 | L |
|  |  | Sleep dependent | 2 | 624.93 | <0.0001 | **** | 10.62 | L |
|  |  | Circadian | 5 | 27.95 | <0.0001 | **** | 0.44 | L |
|  |  | Amplitude threshold*Circadian | 5 | 2.47 | 0.0324 |  | 0.04 | S |
|  |  | Amplitude threshold*Sleep dependent | 2 | 105.44 | <0.0001 | **** | 1.79 | L |
|  |  | Sleep dependent*Circadian | 10 | 4.78 | <0.0001 | **** | 0.09 | S |
| Duration | Initial | Amplitude threshold | 1 | 5360.31 | <0.0001 | **** | 40.63 | L |
|  |  | Sleep dependent | 2 | 50.2 | <0.0001 | **** | 0.83 | L |
|  |  | Circadian | 5 | 5.52 | <0.0001 | **** | 0.09 | S |
|  |  | Amplitude threshold*Circadian | 5 | 5.14 | 0.0002 | *** | 0.08 | S |
|  |  | Amplitude threshold*Sleep dependent | 2 | 12.8 | <0.0001 | **** | 0.21 | M |
|  |  | Sleep dependent*Circadian | 10 | 5.83 | <0.0001 | **** | 0.11 | S |
|  | Final | Amplitude threshold | 1 | 3897.31 | <0.0001 | **** | 30.36 | L |
|  |  | Sleep dependent | 2 | 12.28 | <0.0001 | **** | 0.21 | M |
|  |  | Circadian | 5 | 9.69 | <0.0001 | **** | 0.17 | M |
|  |  | Amplitude threshold*Circadian | 5 | 6.55 | <0.0001 | **** | 0.10 | S |
|  |  | Amplitude threshold*Sleep dependent | 2 | 29.64 | <0.0001 | **** | 0.50 | L |
|  |  | Sleep dependent*Circadian | 10 | 4.16 | <0.0001 | **** | 0.08 | S |
| Mean Slope | Initial | Amplitude threshold | 1 | 13508.1 | <0.0001 | **** | 101.74 | L |
|  |  | Sleep dependent | 2 | 335.98 | <0.0001 | **** | 5.65 | L |
|  |  | Circadian | 5 | 36.78 | <0.0001 | **** | 0.59 | L |
|  |  | Amplitude threshold*Circadian | 5 | 0.58 | ns |  |  |  |
|  |  | Amplitude threshold*Sleep dependent | 2 | 74.95 | <0.0001 | **** | 1.26 | L |
|  |  | Sleep dependent*Circadian | 10 | 2.33 | 0.011 |  | 0.04 | S |
|  | Final | Amplitude threshold | 1 | 8979.5 | <0.0001 | **** | 73.57 | L |
|  |  | Sleep dependent | 2 | 330.59 | <0.0001 | **** | 5.77 | L |
|  |  | Circadian | 5 | 42.85 | <0.0001 | **** | 0.76 | L |
|  |  | Amplitude threshold*Circadian | 5 | 1.13 | ns |  |  |  |
|  |  | Amplitude threshold*Sleep dependent | 2 | 43.05 | <0.0001 | **** | 0.75 | L |
|  |  | Sleep dependent*Circadian | 10 | 1.58 | ns |  |  |  |
| Maximum | Initial | Amplitude threshold | 1 | 13469.6 | <0.0001 | **** | 101.55 | L |
| slope |  | Sleep dependent | 2 | 402.52 | <0.0001 | **** | 6.77 | L |
|  |  | Circadian | 5 | 33.97 | <0.0001 | **** | 0.53 | L |
|  |  | Amplitude threshold*Circadian | 5 | 0.42 | ns |  |  |  |
|  |  | Amplitude threshold*Sleep dependent | 2 | 60.43 | <0.0001 | **** | 1.02 | L |
|  |  | Sleep dependent*Circadian | 10 | 3.09 | 0.0008 | ** | 0.06 | S |
|  | Final | Amplitude threshold | 1 | 12797.8 | <0.0001 | **** | 105.06 | L |
|  |  | Sleep dependent | 2 | 470.75 | <0.0001 | **** | 7.98 | L |
|  |  | Circadian | 5 | 45.73 | <0.0001 | **** | 0.75 | L |
|  |  | Amplitude threshold*Circadian | 5 | 0.79 | ns |  |  |  |
|  |  | Amplitude threshold*Sleep dependent | 2 | 39.04 | <0.0001 | **** | 0.66 | L |
|  |  | Sleep dependent*Circadian | 10 | 2.75 | 0.003 | * | 0.05 | S |

Results for negative half-waves are presented. The amplitude threshold factor comprises the < -5 µV and the < -37.5 µV detection thresholds. The sleep-dependent factor includes thirds of the total sleep period (9h20m). The circadian factor comprises 6*60 degree bins. The Segment variable indicates the descending (initial) or the ascending (final) phase of the slow wave (SW) negative half waves. Degree of freedom (DF), *F* values, *P* values, effect size (*Cohen’s f ^2^*) of main effects, and interactions are indicated for each studied variables as returned from mixed model analyses of variances ( * *P* < .005, ** *P* < .001, *** *P* < .0005, **** *P* <.0001). Superscripts following effect size values indicate the magnitude of the effects size [small(S): 0.02-0.15, medium (M): 0.15-0.35, large (L): >0.35]. *P* values and effect sizes for non-significant effects are not indicated. Non-significant trends (<0.05) are indicated.
