# Supplementary material for: Circadian regulation of slow waves in human sleep: Topographical aspects
Source: Neuroimage. 2015 Aug 1;116:123–34. doi: 10.1016/j.neuroimage.2015.05.012 (PMC4503801; doi:10.1016/j.neuroimage.2015.05.012)
Supplement: Inline Supplementary Table S6 [file mmc6.doc]

**Table S6. Summary of main effects and interactions of the frequency range, sleep dependent and circadian factors on the studied SW parameters as measured during the forced desynchrony**

| SW parameter | Segment | Effect | *DF* | *F* value | *P* value |  | Cohen's *f ^2^* |  |
| --- | --- | --- | --- | --- | --- | --- | --- | --- |
| Incidence |  | Frequency range | 1 | 22.3 | <0.0001 | **** | 0.25 | M |
|  |  | Sleep dependent | 2 | 1119.14 | <0.0001 | **** | 18.45 | L |
|  |  | Circadian | 5 | 43 | <0.0001 | **** | 0.63 | L |
|  |  | Frequency range*Circadian | 5 | 0.16 | ns |  |  |  |
|  |  | Frequency range*Sleep dependent | 2 | 2.41 | ns |  |  |  |
|  |  | Sleep dependent*Circadian | 10 | 7.08 | <0.0001 | **** | 0.15 | S |
| Amplitude |  | Frequency range | 1 | 17.16 | <0.0001 | **** | 0.18 | M |
|  |  | Sleep dependent | 2 | 553.75 | <0.0001 | **** | 9.27 | L |
|  |  | Circadian | 5 | 32.64 | <0.0001 | **** | 0.59 | L |
|  |  | Frequency range*Circadian | 5 | 0.07 | ns |  |  |  |
|  |  | Frequency range*Sleep dependent | 2 | 0.37 | ns |  |  |  |
|  |  | Sleep dependent*Circadian | 10 | 5.04 | <0.0001 | **** | 0.11 | S |
| Duration | Initial | Frequency range | 1 | 186 | <0.0001 | **** | 1.63 | L |
|  |  | Sleep dependent | 2 | 45.6 | <0.0001 | **** | 0.68 | L |
|  |  | Circadian | 5 | 9.48 | <0.0001 | **** | 0.15 | S |
|  |  | Frequency range*Circadian | 5 | 0.28 | ns |  |  |  |
|  |  | Frequency range*Sleep dependent | 2 | 0.64 | ns |  |  |  |
|  |  | Sleep dependent*Circadian | 10 | 5.46 | <0.0001 | **** | 0.11 | S |
|  | Final | Frequency range | 1 | 118.15 | <0.0001 | **** | 1.13 | L |
|  |  | Sleep dependent | 2 | 12.52 | <0.0001 | **** | 0.21 | M |
|  |  | Circadian | 5 | 20.02 | <0.0001 | **** | 0.39 | L |
|  |  | Frequency range*Circadian | 5 | 0.34 | ns |  |  |  |
|  |  | Frequency range*Sleep dependent | 2 | 0.27 | ns |  |  |  |
|  |  | Sleep dependent*Circadian | 10 | 3.77 | <0.0001 | **** | 0.08 | S |
| Mean Slope | Initial | Frequency range | 1 | 147.19 | <0.0001 | **** | 1.48 | L |
|  |  | Sleep dependent | 2 | 165.91 | <0.0001 | **** | 2.61 | L |
|  |  | Circadian | 5 | 40.7 | <0.0001 | **** | 0.63 | L |
|  |  | Frequency range*Circadian | 5 | 0.37 | ns |  |  |  |
|  |  | Frequency range*Sleep dependent | 2 | 0.43 | ns |  |  |  |
|  |  | Sleep dependent*Circadian | 10 | 3.5 | 0.0002 | *** | 0.07 | S |
|  | Final | Frequency range | 1 | 82.28 | <0.0001 | **** | 0.87 | L |
|  |  | Sleep dependent | 2 | 185.93 | <0.0001 | **** | 3.38 | L |
|  |  | Circadian | 5 | 46.71 | <0.0001 | **** | 1.03 | L |
|  |  | Frequency range*Circadian | 5 | 0.5 | ns |  |  |  |
|  |  | Frequency range*Sleep dependent | 2 | 0.14 | ns |  |  |  |
|  |  | Sleep dependent*Circadian | 10 | 2.24 | 0.015 |  | 0.05 | S |
| Maximum slope | Initial | Frequency range | 1 | 71.03 | <0.0001 | **** | 0.77 | L |
|  |  | Sleep dependent | 2 | 284.52 | <0.0001 | **** | 4.45 | L |
|  |  | Circadian | 5 | 38.03 | <0.0001 | **** | 0.55 | L |
|  |  | Frequency range*Circadian | 5 | 0.11 | ns |  |  |  |
|  |  | Frequency range*Sleep dependent | 2 | 0.12 | ns |  |  |  |
|  |  | Sleep dependent*Circadian | 10 | 3.53 | 0.0002 | *** | 0.07 | S |
|  | Final | Frequency range | 1 | 56.81 | <0.0001 | **** | 0.62 | L |
|  |  | Sleep dependent | 2 | 381.97 | <0.0001 | **** | 6.58 | L |
|  |  | Circadian | 5 | 57.46 | <0.0001 | **** | 1.07 | L |
|  |  | Frequency range*Circadian | 5 | 0.2 | ns |  |  |  |
|  |  | Frequency range*Sleep dependent | 2 | 0.09 | ns |  |  |  |
|  |  | Sleep dependent*Circadian | 10 | 2.56 | 0.0051 |  | 0.06 | S |

We assessed the effect of the frequency band-width used for SW detection. All SW parameters for the frequency band 0.5-2 Hz and 0.5-4 Hz were analyzed in the same mixed model adding the factor ‘frequency range’. We found this factor to have a significant effect on most SW parameters, however, the sleep-dependent and circadian main effects remained significant and were not modulated by the frequency range used for the analysis (Table S5).

Results for negative half-waves are presented. The frequency range factor comprises the 0.5-2 Hz and the 0.5-4 Hz band-width. The sleep-dependent factor includes thirds of the total sleep period (9 h 20 m). The circadian factor comprises 6*60 degree bins. The Segment variable indicates the descending (initial) or the ascending (final) phase of the slow wave (SW) negative half waves. Degree of freedom (DF), *F* values, *P* values, effect size (*Cohen’s f ^2^*) of main effects, and interactions are indicated for each studied variables as returned from mixed model analyses of variances ( * *P* < .005, ** *P* < .001, *** *P* < .0005, **** *P* <.0001). Superscripts following effect size values indicate the magnitude of the effects size [small(S): 0.02-0.15, medium (M): 0.15-0.35, large (L): >0.35]. *P* values and effect sizes for non-significant effects are not indicated. Non-significant trends (<0.05) are indicated.
