# Supplementary material for: Circadian regulation of slow waves in human sleep: Topographical aspects
Source: Neuroimage. 2015 Aug 1;116:123–34. doi: 10.1016/j.neuroimage.2015.05.012 (PMC4503801; doi:10.1016/j.neuroimage.2015.05.012)
Supplement: Inline Supplementary Table S7 [file mmc7.doc]

**Table S7.** Summary of main effects and interactions of EEG reference, sleep dependent and circadian factors on the studied SW parameters as measured during the forced desynchrony

| SW parameter | Segment | Effect | *DF* | *F* value | *P* value |  | Cohen's *f^2^* |  |
| --- | --- | --- | --- | --- | --- | --- | --- | --- |
| Incidence |  | EEG reference | 1 | 313.98 | <0.0001 | **** | 3.19 | L |
|  |  | Sleep dependent | 2 | 1094.29 | <0.0001 | **** | 17.78 | L |
|  |  | Circadian | 5 | 45.47 | <0.0001 | **** | 0.67 | L |
|  |  | EEG reference*Sleep dependent | 2 | 24.31 | <0.0001 | **** | 0.40 | L |
|  |  | EEG reference*Circadian | 5 | 1.6 | ns |  | 0.02 | S |
|  |  | Sleep dependent*Circadian | 10 | 6.19 | <0.0001 | **** | 0.13 | S |
| Amplitude |  | EEG reference | 1 | 89.49 | <0.0001 | **** | 0.75 | L |
|  |  | Sleep dependent | 2 | 352.32 | <0.0001 | **** | 5.86 | L |
|  |  | Circadian | 5 | 33.41 | <0.0001 | **** | 0.58 | L |
|  |  | EEG reference*Sleep dependent | 2 | 0.22 | ns |  |  |  |
|  |  | EEG reference*Circadian | 5 | 0.74 | ns |  |  |  |
|  |  | Sleep dependent*Circadian | 10 | 4.92 | <0.0001 | **** | 0.10 | S |
| Duration | Initial | EEG reference | 1 | 1.65 | ns |  |  |  |
|  |  | Sleep dependent | 2 | 25.57 | <0.0001 | **** | 0.42 | L |
|  |  | Circadian | 5 | 17.78 | <0.0001 | **** | 0.28 | M |
|  |  | EEG reference*Sleep dependent | 2 | 17.81 | <0.0001 | **** | 0.29 | M |
|  |  | EEG reference*Circadian | 5 | 1.99 | ns |  |  |  |
|  |  | Sleep dependent*Circadian | 10 | 4.87 | <0.0001 | **** | 0.09 | S |
|  | Final | EEG reference | 1 | 362.36 | <0.0001 | **** | 2.94 | L |
|  |  | Sleep dependent | 2 | 10.77 | <0.0001 | **** | 0.18 | M |
|  |  | Circadian | 5 | 22.95 | <0.0001 | **** | 0.39 | L |
|  |  | EEG reference*Sleep dependent | 2 | 1.47 | ns |  |  |  |
|  |  | EEG reference*Circadian | 5 | 0.22 | ns |  |  |  |
|  |  | Sleep dependent*Circadian | 10 | 4.2 | <0.0001 | **** | 0.08 | S |
| Mean Slope | Initial | EEG reference | 1 | 54.12 | <0.0001 | **** | 0.47 | L |
|  |  | Sleep dependent | 2 | 175.86 | <0.0001 | **** | 2.86 | L |
|  |  | Circadian | 5 | 61.83 | <0.0001 | **** | 0.95 | L |
|  |  | EEG reference*Sleep dependent | 2 | 7.37 | 0.0009 | ** | 0.12 | S |
|  |  | EEG reference*Circadian | 5 | 1.79 | ns |  |  |  |
|  |  | Sleep dependent*Circadian | 10 | 3.45 | 0.0002 | *** | 0.07 | S |
|  | Final | EEG reference | 1 | 320.74 | <.0001 | **** | 2.89 | L |
|  |  | Sleep dependent | 2 | 116.82 | <.0001 | **** | 2.00 | L |
|  |  | Circadian | 5 | 56.63 | <.0001 | **** | 1.08 | L |
|  |  | EEG reference*Sleep dependent | 2 | 0.96 | ns |  |  |  |
|  |  | EEG reference*Circadian | 5 | 0.94 | ns |  |  |  |
|  |  | Sleep dependent*Circadian | 10 | 2.84 | 0.0019 | * | 0.06 | S |
| Maximum slope | Initial | EEG reference | 1 | 6.56 | 0.0117 |  | 0.06 | S |
|  |  | Sleep dependent | 2 | 236.03 | <0.0001 | **** | 3.82 | L |
|  |  | Circadian | 5 | 57.1 | <0.0001 | **** | 0.85 | L |
|  |  | EEG reference*Sleep dependent | 2 | 3.34 | 0.0388 |  | 0.05 | S |
|  |  | EEG reference*Circadian | 5 | 0.99 | ns |  |  |  |
|  |  | Sleep dependent*Circadian | 10 | 3.41 | 0.0002 | *** | 0.07 | S |
|  | Final | EEG reference | 1 | 201.94 | <0.0001 | **** | 1.85 | L |
|  |  | Sleep dependent | 2 | 219.44 | <0.0001 | **** | 3.69 | L |
|  |  | Circadian | 5 | 67.17 | <0.0001 | **** | 1.18 | L |
|  |  | EEG reference*Sleep dependent | 2 | 2.51 | ns |  |  |  |
|  |  | EEG reference*Circadian | 5 | 0.8 | ns |  |  |  |
|  |  | Sleep dependent*Circadian | 10 | 2.38 | 0.0092 |  | 0.05 | S |
|  |  |  |  |  |  |  |  |  |

We investigated the influence of the EEG reference (common reference *versus* contralateral mastoid reference) used for SW analyses on the sleep dependent and circadian variation of SW parameters. The factor ‘EEG reference’ had a significant main effect on most studied SW parameters except the duration of the initial segment of SW half waves independent of the sleep dependent and circadian factors which remained strongly significant. Whereas the EEG reference significantly modulated the sleep dependent variation of some SW parameters such as the incidence, amplitude, as well as the duration, and slope of the initial segments of the half waves, the circadian regulation of SWs was not modulated by the EEG reference.

Results for negative half-waves are presented. The EEG reference factor comprises the common reference (where all electrodes are referenced to Pz) and the contralateral mastoid reference (where all EEG electrodes from a certain brain hemisphere are referenced to the mastoid area of the opposite hemisphere: A1 and A2). The sleep-dependent factor includes thirds of the total sleep period (9 h 20 m). The circadian factor comprises 6*60 degree bins. The Segment variable indicates the descending (initial) or the ascending (final) phase of the slow wave (SW) negative half waves. Degree of freedom (DF), *F* values, *P* values, effect size (*Cohen’s f ^2^*) of main effects, and interactions are indicated for each studied variables as returned from mixed model analyses of variances ( * *P* < .005, ** *P* < .001, *** *P* < .0005, **** *P* <.0001). Superscripts following effect size values indicate the magnitude of the effects size [small(S): 0.02-0.15, medium (M): 0.15-0.35, large (L): >0.35]. *P* values and effect sizes for non-significant effects are not indicated. Non-significant trends (<0.05) are indicated.
