# Supplementary material for: Circadian regulation of slow waves in human sleep: Topographical aspects
Source: Neuroimage. 2015 Aug 1;116:123–34. doi: 10.1016/j.neuroimage.2015.05.012 (PMC4503801; doi:10.1016/j.neuroimage.2015.05.012)
Supplement: Inline Supplementary Table S8 [file mmc8.doc]

**Table S8.** Summary of main effects and interactions of SW half wave polarity, sleep dependent and circadian factors on the studied SW parameters as measured during the forced desynchrony

| SW parameter | Segment | 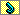Effect | *DF* | *F* value | *P* value |  | Cohen’s *f ^2^* |  |
| --- | --- | --- | --- | --- | --- | --- | --- | --- |
| Incidence |  | Polarity | 1 | 0.04 | ns |  |  |  |
|  |  | Sleep dependent | 2 | 1024.41 | <0.0001 | **** | 17.03 | L |
|  |  | Circadian | 5 | 44.51 | <0.0001 | **** | 0.65 | L |
|  |  | Polarity*Circadian | 5 | 0.01 | ns |  |  |  |
|  |  | Polarity*Sleep dependent | 2 | 0.07 | ns |  |  |  |
|  |  | Sleep dependent*Circadian | 10 | 7.34 | <0.0001 | **** | 0.16 | M |
| Amplitude |  | Polarity | 1 | 8.64 | 0.004 | * | 0.08 | S |
|  |  | Sleep dependent | 2 | 440.31 | <0.0001 | **** | 7.19 | L |
|  |  | Circadian | 5 | 23.43 | <0.0001 | **** | 0.41 | L |
|  |  | Polarity*Circadian | 5 | 0.08 | ns |  |  |  |
|  |  | Polarity*Sleep dependent | 2 | 0.08 | ns |  |  |  |
|  |  | Sleep dependent*Circadian | 10 | 4.6 | <0.0001 | **** | 0.10 | S |
| Duration | Initial | Polarity | 1 | 0.47 | ns |  |  |  |
|  |  | Sleep dependent | 2 | 32.11 | <0.0001 | **** | 0.50 | L |
|  |  | Circadian | 5 | 11.3 | <0.0001 | **** | 0.18 | M |
|  |  | Polarity*Circadian | 5 | 0.18 | ns |  |  |  |
|  |  | Polarity*Sleep dependent | 2 | 0.34 | ns |  |  |  |
|  |  | Sleep dependent*Circadian | 10 | 4.13 | <0.0001 | **** | 0.08 | S |
|  | Final | Polarity | 1 | 21.2 | <0.0001 | **** | 0.16 | M |
|  |  | Sleep dependent | 2 | 11.84 | <0.0001 | **** | 0.20 | M |
|  |  | Circadian | 5 | 8.97 | <0.0001 | **** | 0.15 | S |
|  |  | Polarity*Circadian | 5 | 0.19 | ns |  |  |  |
|  |  | Polarity*Sleep dependent | 2 | 2.92 | ns |  |  |  |
|  |  | Sleep dependent*Circadian | 10 | 3.69 | <0.0001 | **** | 0.07 | S |
| Mean Slope | Initial | Polarity | 1 | 0.47 | ns |  |  |  |
|  |  | Sleep dependent | 2 | 123.33 | <0.0001 | **** | 1.94 | L |
|  |  | Circadian | 5 | 35.57 | <0.0001 | **** | 0.54 | L |
|  |  | Polarity*Circadian | 5 | 0.2 | ns |  |  |  |
|  |  | Polarity*Sleep dependent | 2 | 0.03 | ns |  |  |  |
|  |  | Sleep dependent*Circadian | 10 | 3.76 | <0.0001 | **** | 0.07 | S |
|  | Final | Polarity | 1 | 14.22 | 0.0003 | *** | 0.12 | S |
|  |  | Sleep dependent | 2 | 94.34 | <0.0001 | **** | 1.59 | L |
|  |  | Circadian | 5 | 22.65 | <0.0001 | **** | 0.41 | L |
|  |  | Polarity*Circadian | 5 | 0.57 | ns |  |  |  |
|  |  | Polarity*Sleep dependent | 2 | 2.68 | ns |  |  |  |
|  |  | Sleep dependent*Circadian | 10 | 2.65 | 0.0037 | * | 0.05 | S |
| Maximum slope | Initial | Polarity | 1 | 12.6 | 0.0006 | ** | 0.12 | S |
|  |  | Sleep dependent | 2 | 197.96 | <0.0001 | **** | 3.16 | L |
|  |  | Circadian | 5 | 40.39 | <0.0001 | **** | 0.61 | L |
|  |  | Polarity*Circadian | 5 | 0.22 | ns |  |  |  |
|  |  | Polarity*Sleep dependent | 2 | 0.55 | ns |  |  |  |
|  |  | Sleep dependent*Circadian | 10 | 3.58 | 0.0001 | **** | 0.07 | S |
|  | Final | Polarity | 1 | 5.9 | 0.0168 |  | 0.05 | S |
|  |  | Sleep dependent | 2 | 252.94 | <0.0001 | **** | 4.14 | L |
|  |  | Circadian | 5 | 33.58 | <0.0001 | **** | 0.58 | L |
|  |  | Polarity*Circadian | 5 | 0.56 | ns |  |  |  |
|  |  | Polarity*Sleep dependent | 2 | 1.26 | ns |  |  |  |
|  |  | Sleep dependent*Circadian | 10 | 2.67 | 0.0035 | * | 0.05 | S |

We assessed the effect of polarity on the studied SW parameters in addition to the circadian and sleep-dependent factors. We found that factor ‘polarity’ had an independent significant effect on most SW parameters except incidence, duration and mean slope of the initial segment and the maximum slope of the final segment of SW half-waves. However, overall the effect size of polarity was smaller as compared to the sleep-dependent and the circadian regulation, which yielded strong significance and no significant interaction with polarity. The only exception was the number of peaks per SW half-wave, which showed a very stronger polarity effect. The positive half-waves had significantly more peaks.

The polarity factor includes the SW parameters for the negative and positive half-waves. The sleep-dependent factor includes thirds of the total sleep period (9 h 20 m). The circadian factor comprised 6*60 degree bins. The Segment variable indicates the descending (initial) or the ascending (final) phase of the slow wave (SW) negative half waves. Degree of freedom (DF), *F* values, *P* values, effect size (*Cohen’s f ^2^*) of main effects, and interactions are indicated for each studied variables as returned from mixed model analyses of variances ( * *P* < .005, ** *P* < .001, *** *P* < .0005, **** *P* <.0001). Superscripts following effect size values indicate the magnitude of the effects size [small(S): 0.02-0.15, medium (M): 0.15-0.35, large (L): >0.35]. *P* values and effect sizes for non-significant effects are not indicated. Non-significant trends (<0.05) are indicated.
