# Supplementary material for: Circadian regulation of slow waves in human sleep: Topographical aspects
Source: Neuroimage. 2015 Aug 1;116:123–34. doi: 10.1016/j.neuroimage.2015.05.012 (PMC4503801; doi:10.1016/j.neuroimage.2015.05.012)
Supplement: Inline Supplementary Table S9 [file mmc9.doc]

**Table S9. Summary of main effects and interactions of the SW half wave segment, sleep dependent and circadian factors on the studied SW parameters as measured during the forced desynchrony**

| Polarity | SW parameter | Effect | *DF* | *F* value | *P* value |  | Cohen's *f ^2^* |  |
| --- | --- | --- | --- | --- | --- | --- | --- | --- |
| Negative | Duration | Segment | 1 | 31.7 | <0.0001 | **** | 0.26 | M |
|  |  | Sleep dependent | 2 | 14.8 | <0.0001 | **** | 0.24 | M |
|  |  | Circadian | 5 | 14.63 | <0.0001 | **** | 0.25 | M |
|  |  | Segment*Circadian | 5 | 0.26 | ns |  |  |  |
|  |  | Segment*Sleep dependent | 2 | 9.41 | 0.0002 | *** | 0.15 | S |
|  |  | Sleep dependent*Circadian | 10 | 4.07 | <0.0001 | **** | 0.08 | S |
|  | Mean slope | Segment | 1 | 70.87 | <0.0001 | **** | 0.71 | L |
|  |  | Sleep dependent | 2 | 144.53 | <0.0001 | **** | 2.44 | L |
|  |  | Circadian | 5 | 43.22 | <0.0001 | **** | 0.84 | L |
|  |  | Segment*Circadian | 5 | 0.94 | ns |  |  |  |
|  |  | Segment*Sleep dependent | 2 | 5.66 | 0.0045 | * | 0.10 | S |
|  |  | Sleep dependent*Circadian | 10 | 2.49 | 0.007 |  | 0.05 | S |
|  | Maximum slope | Segment | 1 | 82.06 | <0.0001 | **** | 0.79 | L |
|  |  | Sleep dependent | 2 | 259.39 | <0.0001 | **** | 4.22 | L |
|  |  | Circadian | 5 | 45.29 | <0.0001 | **** | 0.74 | L |
|  |  | Segment*Circadian | 5 | 0.58 | ns |  |  |  |
|  |  | Segment*Sleep dependent | 2 | 3.69 | 0.028 |  | 0.06 | S |
|  |  | Sleep dependent*Circadian | 10 | 2.64 | 0.004 | * | 0.05 | S |
| Positive | Duration | Segment | 1 | 1.32 | ns |  |  |  |
|  |  | Sleep dependent | 2 | 23.46 | <0.0001 | **** | 0.38 | L |
|  |  | Circadian | 5 | 7.36 | <0.0001 | **** | 0.11 | S |
|  |  | Segment*Circadian | 5 | 0.16 | ns |  |  |  |
|  |  | Segment*Sleep dependent | 2 | 0.33 | ns |  |  |  |
|  |  | Sleep dependent*Circadian | 10 | 4.03 | <0.0001 | **** | 0.07 | S |
|  | Mean slope | Segment | 1 | 9.43 | 0.003 | * | 0.08 | S |
|  |  | Sleep dependent | 2 | 105.25 | <0.0001 | **** | 1.66 | L |
|  |  | Circadian | 5 | 18.57 | <0.0001 | **** | 0.28 | M |
|  |  | Segment*Circadian | 5 | 0.22 | ns |  |  |  |
|  |  | Segment*Sleep dependent | 2 | 0.35 | ns |  |  |  |
|  |  | Sleep dependent*Circadian | 10 | 3.99 | <0.0001 | **** | 0.08 | S |
|  | Maximum slope | Segment | 1 | 188.79 | <0.0001 | **** | 1.74 | L |
|  |  | Sleep dependent | 2 | 200.75 | <0.0001 | **** | 3.22 | L |
|  |  | Circadian | 5 | 30.42 | <0.0001 | **** | 0.48 | L |
|  |  | Segment*Circadian | 5 | 0.28 | ns |  |  |  |
|  |  | Segment*Sleep dependent | 2 | 0.14 | ns |  |  |  |
|  |  | Sleep dependent*Circadian | 10 | 3.68 | <0.0001 | **** | 0.07 | S |

We assessed the effect of the ascending *versus* descending transitions between the positive and negative half-waves (initial *versus* final segment) for the circadian and sleep-dependent modulation of SW duration and slope measures. We found that both the circadian and sleep-dependent regulations were significantly present independent of the half-wave segment factor, which also yielded a significant effect on the studied SW parameters except for the duration of the positive half-waves. Most importantly, we confirmed that the circadian modulation of SW parameters was totally independent of the segment whereas the sleep-dependent modulation of the negative but not the positive SW half-wave was significantly modulated by the direction of the transition between the polarities.

Results for both negative and positive half-waves are presented. The segment factor of the SW half-waves comprises the *initial* and the *final* segment. The sleep-dependent factor included thirds of the total sleep period (9 h 20 m). The circadian factor comprised 6*60 degree bins. Degree of freedom (DF), *F* values, *P* values, effect size (*Cohen’s f ^2^*) of main effects, and interactions are indicated for each studied variables as returned from mixed model analyses of variances ( * *P* < .005, ** *P* < .001, *** *P* < .0005, **** *P* <.0001). Superscripts following effect size values indicate the magnitude of the effects [small(S): 0.02-0.15, medium (M): 0.15-0.35, large (L): >0.35]. *P* values and effect sizes for non-significant effects are not indicated. Non-significant trends (<0.05) are indicated.
